# mRNA-encoded Cas13 treatment of Influenza via site-specific degradation of genomic RNA

**DOI:** 10.1371/journal.ppat.1012345

**Published:** 2024-07-05

**Authors:** Lorena C. S. Chaves, Nichole Orr-Burks, Daryll Vanover, Varun V. Mosur, Sarah R. Hosking, Pramod Kumar E. K., Hyeyoon Jeong, Younghun Jung, José A. F. Assumpção, Hannah E. Peck, Sarah L. Nelson, Kaitlyn N. Burke, McKinzie A. Garrison, Robert A. Arthur, Henry Claussen, Nicholas S. Heaton, Eric R. Lafontaine, Robert J. Hogan, Chiara Zurla, Philip J. Santangelo

**Affiliations:** 1 Wallace H. Coulter Department of Biomedical Engineering, Emory University and Georgia Institute of Technology, Atlanta, Georgia, United States of America; 2 Department of Infectious Diseases, College of Veterinary Medicine University of Georgia, Athens, Georgia, United States of America; 3 Department of Molecular Genetics and Microbiology, Duke University School of Medicine, Durham, North Carolina, United States of America; 4 Emory Integrated Computational Core, Emory University, Atlanta, Georgia, United States of America; 5 Duke Human Vaccine Institute Duke University School of Medicine, Durham, North Carolina, United States of America; The Ohio State University, UNITED STATES OF AMERICA

## Abstract

The CRISPR-Cas13 system has been proposed as an alternative treatment of viral infections. However, for this approach to be adopted as an antiviral, it must be optimized until levels of efficacy rival or exceed the performance of conventional approaches. To take steps toward this goal, we evaluated the influenza viral RNA degradation patterns resulting from the binding and enzymatic activity of mRNA-encoded LbuCas13a and two crRNAs from a prior study, targeting PB2 genomic and messenger RNA. We found that the genome targeting guide has the potential for significantly higher potency than originally detected, because degradation of the genomic RNA is not uniform across the PB2 segment, but it is augmented in proximity to the Cas13 binding site. The PB2 genome targeting guide exhibited high levels (>1 log) of RNA degradation when delivered 24 hours post-infection *in vitro* and maintained that level of degradation over time, with increasing multiplicity of infection (MOI), and across modern influenza H1N1 and H3N2 strains. Chemical modifications to guides with potent LbuCas13a function, resulted in nebulizer delivered efficacy (>1–2 log reduction in viral titer) in a hamster model of influenza (Influenza A/H1N1/California/04/09) infection given prophylactically or as a treatment (post-infection). Maximum efficacy was achieved with two doses, when administered both pre- and post-infection. This work provides evidence that mRNA-encoded Cas13a can effectively mitigate Influenza A infections opening the door to the development of a programmable approach to treating multiple respiratory infections.

## Author summary

For the CRISPR-Cas13 system to be adopted as an alternative treatment of viral infections, it must reach levels of efficacy that rival or exceed the performance of conventional approaches. We evaluated the degradation patterns resulting from the binding and enzymatic activity of mRNA-encoded LbuCas13a and two previously identified crRNAs, targeting conserved PB2 genomic and messenger RNA sequences.

The genome targeting guide displayed higher potency than originally detected, because RNA degradation was augmented near the guide binding site, leading to 1-log of RNA degradation in vitro, which persisted over time, with increasing multiplicity of infection (MOI), and across modern influenza H1N1 and H3N2 strains. The addition of chemical modifications to the guide resulted in 1-2-log reduction in viral titer upon nebulizer delivery prophylactically or as a treatment in a hamster model of influenza. Maximum efficacy was achieved with two doses administered pre- and post-infection.

## Introduction

The influenza virus is the cause of millions of illnesses every year (seasonal flu) [1] and the cause of four pandemics in the last 100 years (1918, 1957, 1968 and 2009) [2,3]. Major efforts have been made to develop new vaccines and treatments, because of the constant threat of a new pandemic [4]. Currently available vaccines have variable effectiveness, and protection depends on the similarity of the strains used in the vaccine formulations to those in circulation [1]. Recently, mRNA technology was successfully applied in the production of vaccines against COVID-19 [5,6], and new vaccine formulations against flu are being investigated [7–9]. Even if this technology offers several advantages over traditional methods, vaccine-induced immunity decays substantially months after immunization [10,11]. This requires additional doses to maintain protection against the infection [12]. Current drug development against influenza is focused on small molecules and neutralizing antibodies, which require high doses (1–2 g) or frequent re-dosing (multiple times per day for multiple days) to obtain functional outcomes, and they are often plagued by strain limitations (influenza A/B) and antigenic changes [13]. In addition, oseltamivir, favipiravir, pimodivir and baloxavir marboxil have been shown to induce development of resistance over time, with significant (>20 fold) decreases in efficacy against multiple influenza strains [14–16]. The emergence of resistant influenza strains calls for the need for higher efficiency and broader spectrum antivirals that can overcome these limitations.

Since viral RNA-targeting approaches provide a gene-specific therapy to mitigate the rapid evolution of influenza A virus (IAV), here we propose a broad Cas13a mRNA-based antiviral against this pathogen. The type VI CRISPR-Cas13a is an RNA-dependent RNase that is activated when the target RNA (trRNA) complements with a crRNA (guide) and triggers specific and directed RNA cleavage [17,18]. Cas13’s activity does not depend on endogenous nucleases, which may be downregulated or inactivated by viral proteins [19], and once activated, allows for continuous cutting until it is degraded, without the need for re-binding to the RNA target. Over the last few years, this platform has demonstrated efficacy *in vitro and in vivo* against various viral pathogens [20,21], and it represents an innovative and programmable approach that deserves further optimization to achieve levels of efficacy commensurate with widescale adoption.

In Blanchard et al. [22], using A/H1N1/WSN/1933 as a model virus, we provided an initial demonstration of viral knockdown (KD) in A549 cells by concurrent expression of mRNA encoded LbuCas13a (for cytosolic expression) and LbuCas13a-NLS (for nuclear expression). After identifying a sequence at the 5’end of the PB2 segment, conserved across over 51,000 Influenza strains, genome and mRNA targeting guides complementary to this region demonstrated > 50% viral KD by qPCR. Initial proof of efficacy *in vivo* was demonstrated in the mouse model of infection, by delivering mRNA-encoded LbuCas13a+LbuCas13a-NLS and guides to the lung via a vibrating mesh nebulizer. Using the same delivery platform and replacing the crRNA with one targeting the SARS-CoV-2 N gene, mitigation of SARS-CoV-2 was achieved in the hamster model of infection, consistent with Cas13’s modular/programmable nature. The mRNA platform, which was used in all *in vitro* and *in vivo* experiments, yielded transient expression with minimal innate responses and no toxicity.

**Fig 1:**
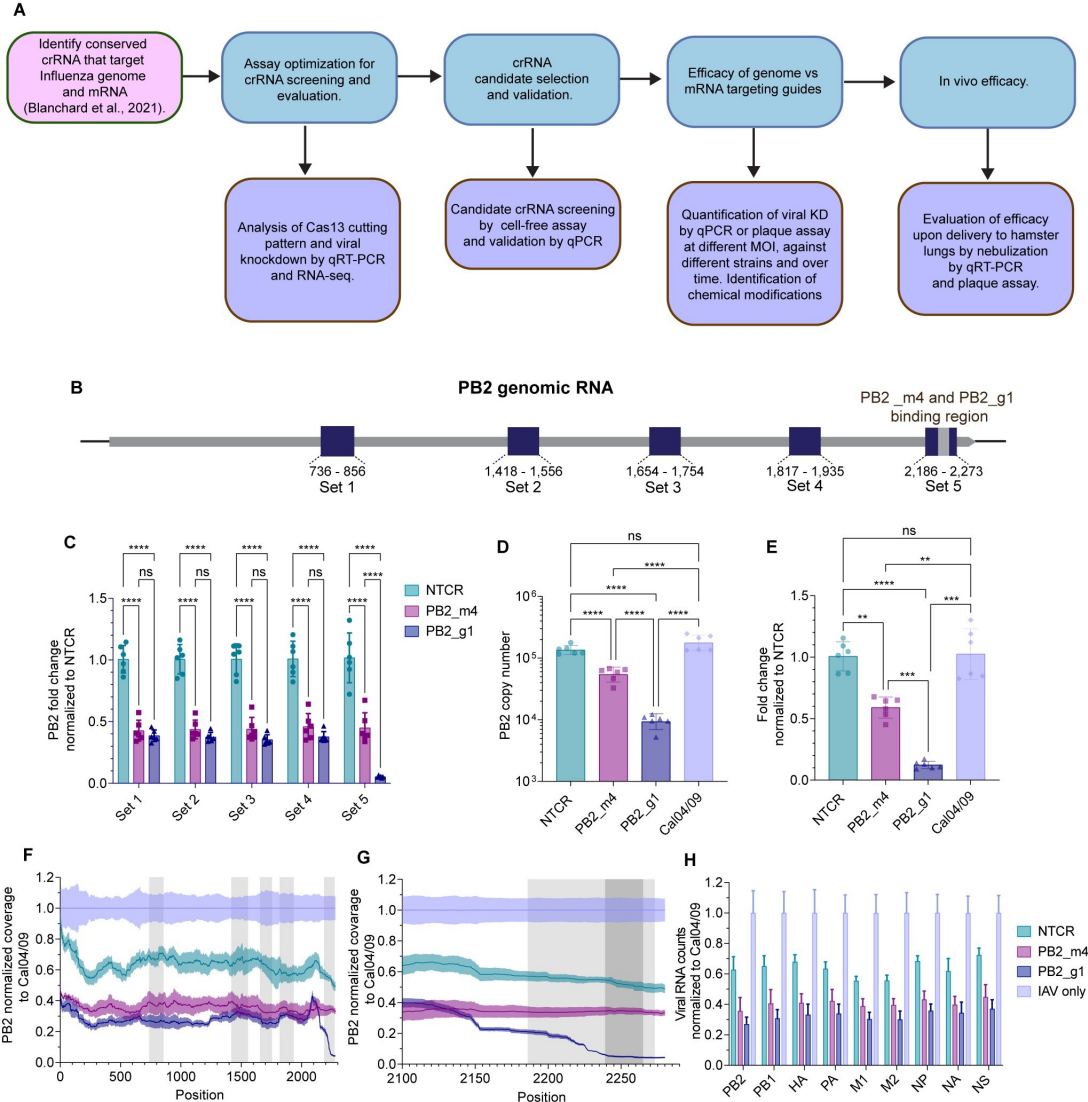
Experimental workflow and crRNA assay optimization analysis. (A) Schematic representation of the experimental workflow. (B) Schematic representation of Influenza PB2 gene (3’ to 5’) with the positions of the qPCR primer/probe Sets 1–5 (dark blue) and crRNA biding site (light grey). (C) Fold change of PB2 RNA levels normalized to the NTCR condition in A549 cells infected with A/H1N1/California/04/09 MOI 0.001 and treated with Cas13 mRNA and the indicated guide using primer/probe Sets 1–5. (D) Copy number of PB2 RNA in A549 cells infected with A/H1N1/California/04/09 MOI 0.001 and treated with Cas13 mRNA and the indicated guide using primer/probe Set 5. (E) Fold change of PB2 RNA levels normalized to NTCR condition for the data in part (B). In parts (C-E), bars represent mean ± s.d. n = 6 per condition and ****p< 0.0001 (Two-way ANOVA with Tukey’s multiple comparisons). p-values for each comparison in part (E) are listed in S4 Table. (F) Coverage of RNA-Seq reads for the A/H1N1/California/04/09 PB2 segment normalized to the coverage of the virus only condition at each position. The sequence amplified by all primer/probe sets is indicated in light grey. (G) Detail of RNA-Seq reads between positions 2100 and 2280. The sequence amplified by primer/probe set 5 is indicated in light grey and the crRNA’s binding site is indicated in dark grey. (H) Counts for all A/H1N1/California/04/09 segments normalized to the virus only condition. The error bands represent mean± SEM (n = 3 per condition). *p*-values for each comparison are listed in S7 Table. Fig 1A and 1B were created in part using BioRender.com.

Here, we sought to further characterize Cas13-based mitigation of influenza infections, focusing on A/H1N1/California/04/09, a more relevant strain, and re-evaluating the potency of one mRNA (PB2_m4) and one genome (PB2_g1) targeting guide, through a rationally designed set of experiments in preparation for an *in vivo* challenge against IAV (Fig 1A). RT-qPCR analysis at multiple sites along the target RNA and RNA-Seq indicated that cleavage of the PB2 genomic sequence by Cas13 led to rapid degradation of the 5’ end of the segment in proximity to its binding site, in comparison to much slower degradation of the 3’ end. This revealed significantly more potent KD by the PB2 genome targeting guide than previously reported. Indeed, while targeting PB2 mRNA was effective, targeting IAV genome provided a consistent and highly potent effect against several H1N1 and H3N2 strains and across multiplicities of infection (MOI).

After characterizing the infection with A/H1N1/California/04/09 in a hamster animal model [23], we optimized the formulation of Cas13 and guide PB2_g1 with the poly(β-amino-thio-ester) polymer (PBATE) P76 for lung delivery via nebulization. This approach demonstrated expression of different cargos, in several animal species, both within the upper respiratory tract (nasopharynx and trachea) and lower respiratory tract, with no detectable short or long-term toxicity [24,25]. In challenge experiments in hamster, Cas13 demonstrated potent mitigation of A/H1N1/California/04/09 infection when delivered before and/or after infection, with one or two doses, achieving 1-2-logs of KD at the level of viral titer. If we are to truly develop this platform as an antiviral, in competition with small molecules and antibodies, high levels of KD of these pathogens must be achieved with minimal dosing.

## Results

### LbuCas13a and guide PB2_g1 induce potent degradation of viral genomic RNA

We previously identified a sequence at the 5’end of the PB2 segment highly conserved across over 51,000 Influenza strains, that have circulated in the last 100 years and including approximately 99.1% of H1N1, H2N2, H3N2, and numerous H5Nx, H7Nx, and H9N2. Guides (crRNAs) against either this PB2 genomic or the mRNA sequence were designed and screened, demonstrating > 50% viral KD by qPCR [22]. To re-evaluate LbuCas13a cutting and viral RNA target degradation by two of these guides, PB2_m4 or PB2_g1 (targeting mRNA and genomic sequences, respectively, S1 Table), A549 cells were infected with influenza A/H1N1/California/04/09 (Cal04/09) at an MOI of 0.001. Twenty-four hours later, cells were transfected with a combination of mRNA encoding for LbuCas13a and LbuCas13a-NLS, for concurrent expression of both the cytosolic and nuclear localizing versions of the RNase (referred to as Cas13 from now onwards) and guides. Delivery of an untargeted (NTCR) guide was used as a control condition. Twenty-four hours post-transfection (hpt) the cells were processed for total RNA extraction and qRT-PCR. Five primer-probe sets were designed to evaluate KD by qPCR. One of these primer-probe sets, set 5, was designed to specifically amplify the region straddling the guide binding site, which is located at the 5’ end of PB2 within the conserved sequence. The other primer sets (Sets 1–4) were located at various distances from the guide’s binding site (Fig 1B and S2 Table). Set 1 was used in our previous work. Results were reported as PB2 fold change using GAPDH as an endogenous control, as its expression was not significantly affected by the treatment (S3 Table). Using primer probe set 1–4, we observed ~55–65% KD of PB2 upon delivery of either guide PB2_m4 or PB2_g1. Surprisingly, using primer probe set 5, we observed ~55% KD with guide PB2_m4 but over 90% KD using guide PB2_g1 (Fig 1C). When we compared PB2 KD by guide PB2_g1 and its reverse complementary version, PB2_RC_g1, we observed ~90% and 33% KD, respectively (S1 Fig).

To further investigate the effect on viral KD by Cas13 and guides PB2_m4 or PB2_g1, we included an infection-only control group (Cal04/09). Total RNA was extracted for qPCR, to confirm the previous results using primer probe set 5, and for next-generation sequencing analysis. As expected, by qPCR, we observed ~ 90% KD using guide PB2_g1 and ~50% KD with guide PB2_m4 (Fig 1D and 1E, and S4 Table). The total RNA was processed for rRNA depletion, cDNA library preparation, and Illumina RNA sequencing. The absolute coverage of the whole PB2 RNA segment in each treatment group was normalized to the virus only group (Figs 1F and S2, and S5 Table). The normalized coverage for the PB2 segment in the PB2_g1 guide treatment was overall similar to the PB2_m4 guide treatment between position 0 and 2150. However, between positions 2170 and 2280, which span Cas13’s binding region, the PB2_g1 guide treatment induced a dramatic decrease in the normalized counts, consistent with the qPCR results (Fig 1G). This suggests that the conserved PB2 genomic sequence is preferentially cut or degraded in close proximity to Cas13’s binding site. Delivery of the NTCR guide induced a significant decrease in PB2 coverage, likely due to previously reported effects of transfection on influenza infection *in vitro* [22]. Last, we analyzed the effect of our treatment on all influenza A segments. Treatment with guide PB2_g1 consistently induced higher KD than PB2_m4, even if not statistically significant, across all segments (Figs 1H and S3 and S4, and S6 and S7 Tables). Therefore, targeting the influenza A genome, in specific locations, rather than the mRNA, provided a more potent reduction of viral RNA than originally detected.

### Screening of additional genome targeting guides

**Fig 2:**
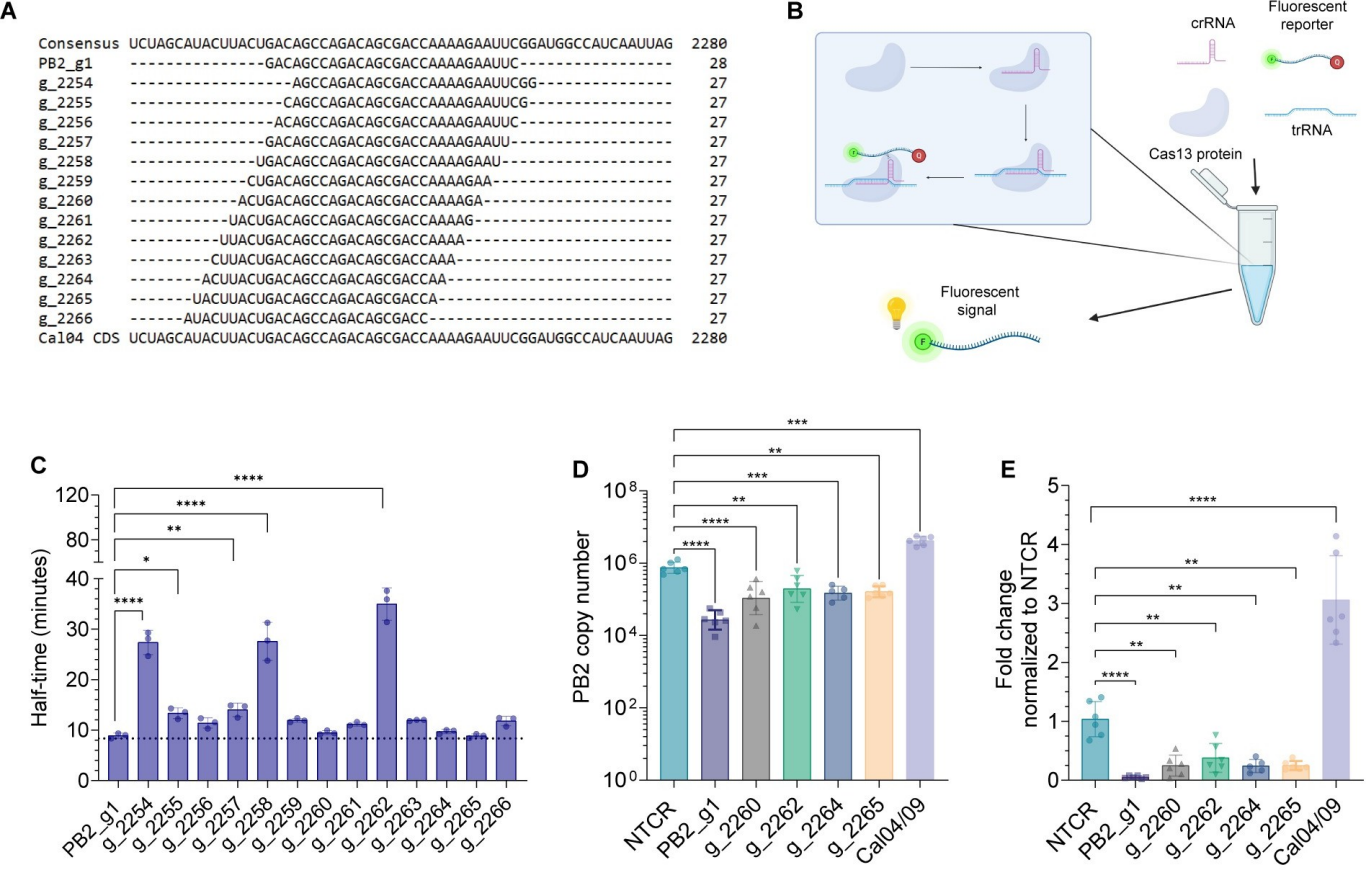
Screening of genome targeting guides across PB2. (A) Sequence of crRNA spacer sequences (5’-3’, S2 Table) tiled across the conserved PB2 genomic sequence. (B) Schematic representation of the cell-free detection assay used to screen the Influenza genome guides in (A) and determine cutting kinetics. (C) Half-time analysis of the raw fluorescence reads generated for each candidate guide by the cell-free assay. The bars represent mean ± s.d. n = 3 per condition. *p = 0.0199, **p =  0.0055, ****p< 0.0001 (Two-way ANOVA with Dunnett’s multiple comparisons) (D) Copy number of PB2 RNA in A549 cells infected with A/H1N1/California/04/09 MOI 0.01 and treated with Cas13 mRNA and the indicated guide using primer/probe Set 5. (E) Fold change of PB2 RNA levels normalized to the NTCR condition for the data in part (D). The bars represent mean ± s.d. n = 6 per condition. *p*-values for each comparison in parts (D and E) are listed in S8 Table. Fig 2B was created in part using BioRender.com.

The genome targeting guides used in our previous work covered a limited region within the identified PB2 conserved sequence [22]. To expand the screen, 13 more guides were designed (Fig 2A and S1 Table) and they were tested using a cell-free assay. The mRNA encoding for LbuCas13a was first translated *in vitro* using a rabbit reticulocyte lysate. The resulting protein was then incubated with each guide, along with a target RNA and a fluorescent reporter molecule. Upon Cas13 activation, the reporter is cleaved, and the resulting increase of fluorescence is recorded over time in a plate reader (Fig 2B). The time required to reach half of the maximum fluorescent signal (half-time) was plotted for each guide. Low half-time values indicate quicker or more efficient Cas13-guide complex formation and faster target RNA degradation. Guides PB2_g1, g_2260, g_2264 and g_2265 displayed similar half-time values, while the half-time for guides g_2254, g_2258 and g_2262 was significantly higher (Figs 2C and S5). PB2 KD induced by a selection of these guides was then evaluated for quantitative analysis by qRT-PCR as described above. The results showed a strong viral RNA knockdown by all candidate genome guides. However, guide PB2_g1 was the most potent, demonstrating > 99% knockdown, while guide g_2262 was the least potent, with ~85% KD (Fig 2D and 2E, and S8 Table). These results demonstrated a strong correlation between the cell-free assay and *in vitro* assay results, indicating that the former can be used for a quick initial evaluation of candidate guides. To understand why guides with sequences shifted by one or few nucleotides would display such differences in potency, we reasoned that crRNA should be designed to have a stable stem-loop structure and targeting regions without substantial base pairing. In our previous work, we used computational folding prediction tools and observed that “well-performing” guides had consistent ΔG  =  −2.74 kcal/mol for the whole sequence and a positive ΔG for the target sequence [22]. In the case of guide PB2_g1, these ΔG values were -2.74 and 0.89 kcal/mol, respectively. Interestingly, guide g_2254 yielded ΔG  =  −2.74 and -2.35 kcal/mol for the whole sequence, predictive of two possible stem-loop structures. Guides g_2259 to g_2266 yielded negative ΔG values for the target sequence (S9 Table). These observations may explain the significant changes in efficacy observed between guides. Last, delivery of Cas13 with or without guide PB2_g1 did not affect cell viability (S6 Fig). Guide PB2_g1 was hence selected for further evaluation *in vitro*.

### Guide PB2_g1 provides sustained mitigation of influenza infection across MOI

**Fig 3:**
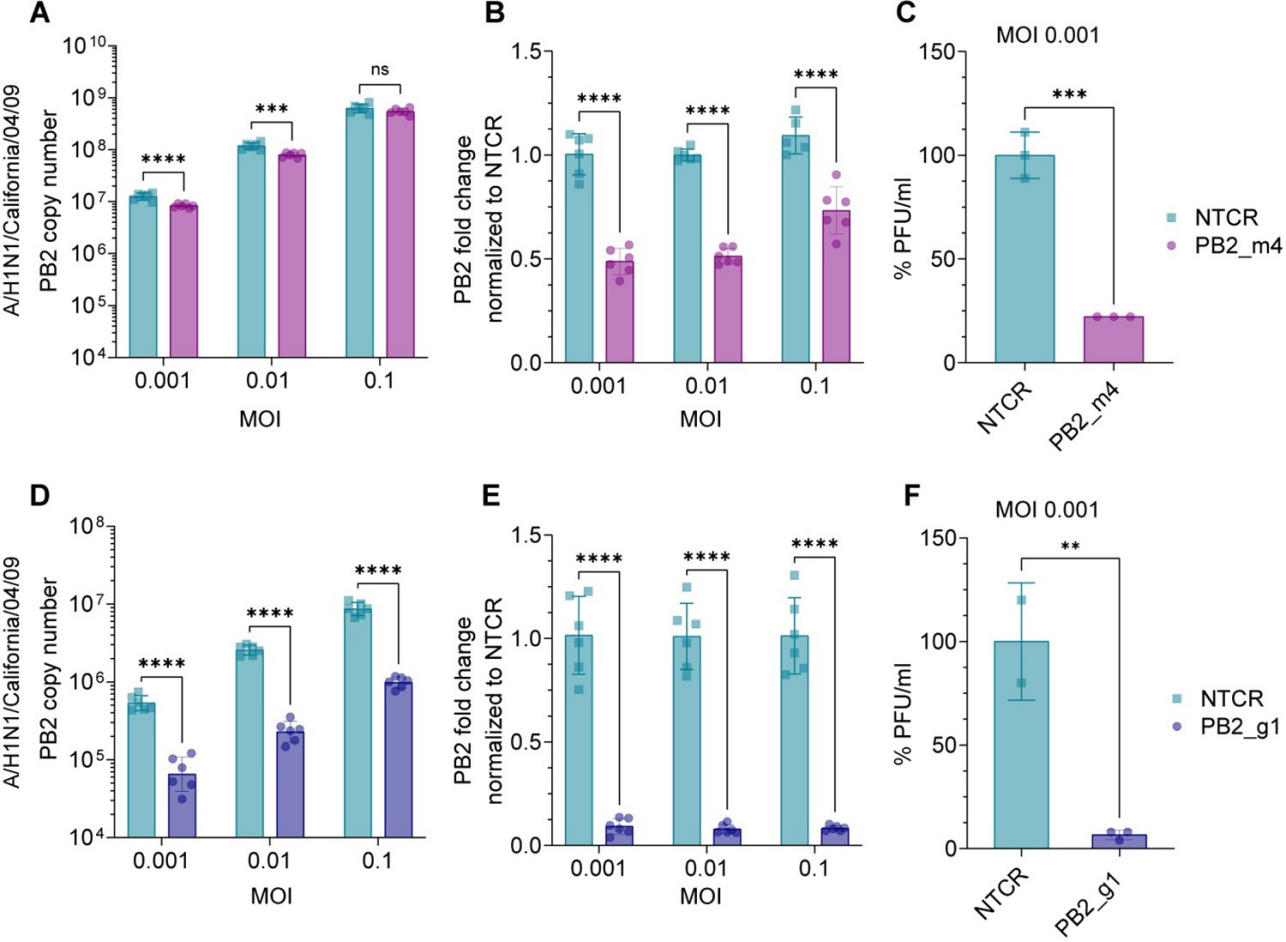
Potency of PB2 mRNA or genome targeting guides across multiplicity of infection. (A) Copy number of PB2 RNA in A549 cells infected with A/H1N1/California/04/09 at the indicated MOIs and treated with Cas13 mRNA and the PB2_m4 mRNA targeting guide using primer/probe Set 5. (B) Fold change of PB2 RNA levels normalized to the NTCR condition for the data in part (A). (C) Viral titer of A/H1N1/California/04/09 after infection at MOI 0.001 and treatment with Cas13a mRNA and guide PB2_m4 or NTCR. The bars represent mean ± s.d. n = 3 per condition. ***p 0.0003 (Unpaired t test comparison). (D) Copy number of PB2 RNA in A549 cells infected with A/H1N1/California/04/09 at the indicated MOIs and treated with Cas13 mRNA and the PB2_g1 genome targeting guide using primer/probe Set 5. (E) Fold change of PB2 RNA levels normalized to the NTCR condition for the data in part (D). (F) Viral titer of A/H1N1/California/04/09 after infection at MOI 0.001 and treatment with Cas13a mRNA and guide PB2_g1 or NTCR. The bars represent mean ± s.d. n = 3 per condition. **p 0.0084 (Unpaired t test comparison). In parts (A,B,D,E) bars represent mean ± s.d. n = 6 per condition. ***p 0.0001 and ****p < 0.0001 (Two-way ANOVA with Šídák’s multiple comparisons).

The initial qRT-PCR and RNA-Seq analysis showed that targeting the viral genome rather than the mRNA may provide more potent mitigation of infection. To test this hypothesis, A549 cells were infected with influenza A/H1N1/California/04/09 at different multiplicity of infection (MOIs); 24h post infection, cells were transfected with mRNA encoded Cas13a, and either guide PB2_m4 or PB2_g1. Twenty-four hours post transfection, the supernatant of the cells was collected for plaque assay and cells were processed for total RNA isolation and qRT-PCR. Treatment with guide PB2_m4 exhibited 52% and 49% knockdown of viral RNA in cells infected with MOI 0.001 and 0.01 respectively, and only 27% in cells infected with MOI 0.1 (Fig 3A and 3B). At MOI 0.001, a 75% decrease of viral titer was measured by plaque assay (Fig 3C). On the contrary, treatment with guide PB2_g1 induced >90% KD of PB2 across all MOIs (Fig 3D and 3E), with >90% decrease in viral titer at MOI 0.001 (Fig 3F). This result confirmed guide PB2_g1 provides consistent and potent mitigation of infection across MOIs, while guide PB2_m4 loses efficacy at high MOIs.

### Cas13’s enzymatic activity is necessary for sustained mitigation of infection across MOI

**Fig 4:**
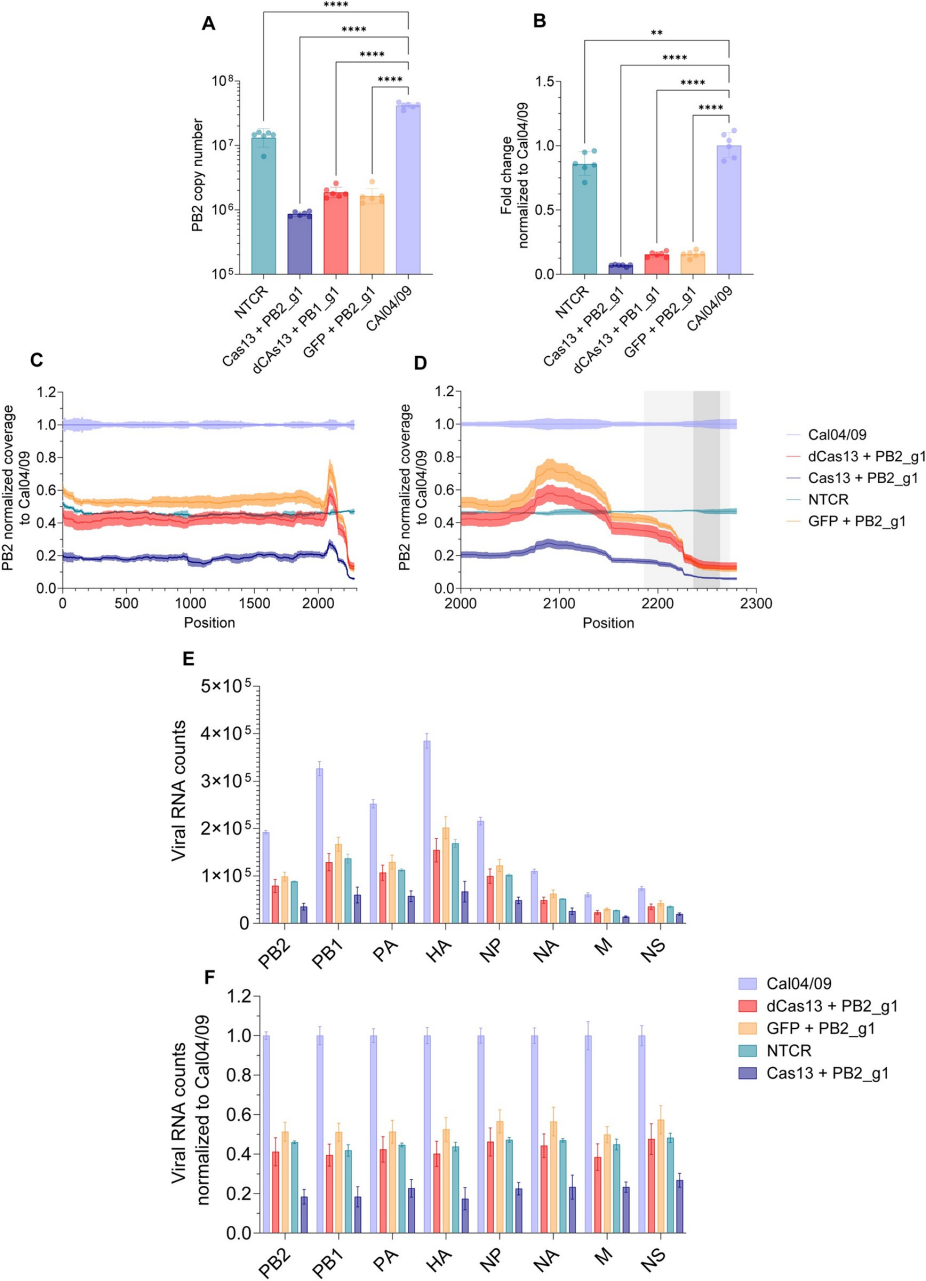
Cas13’s enzymatic activity is needed for potent mitigation of infection. (A) Copy number of PB2 RNA in A549 cells infected with A/H1N1/California/04/09 at MOI 0.1 and treated with Cas13, dCas13 or GFP mRNA and guide PB2_g1 using primer/probe Set 5. (B) Fold change of PB2 RNA levels normalized to the virus only condition for the data in part (A). The bars represent mean ± s.d. n = 6 per condition. ****p < 0.0001 (One-way ANOVA with Tuckey’s multiple comparisons) (C) Coverage of RNA-Seq reads for the A/H1N1/California/04/09 PB2 segment normalized to the coverage of the virus only condition at each position. (D) Detail of RNA-Seq reads between positions 2100 and 2280. The sequence amplified by primer/probe set 5 is indicated in light grey and the crRNA’s binding site is indicated in dark grey. (E) Absolute counts for indicated A/H1N1/California/04/09 segments. (F) Counts normalized to the virus only condition. The error bands represent mean± SEM (n = 3 per condition). *p*-values for each comparison are listed in S11 Table.

To demonstrate that Cas13 enzymatic activity is needed for sustained viral KD, A549 cells were infected with influenza A/H1N1/California/04/09 at MOI 0.1; 24h post infection, cells were transfected with mRNA encoded Cas13, or a deactivated version of Cas13 (dCas13, which binds to the target region but lacks RNAse activity) or mRNA encoded GFP, and guide PB2_g1. Twenty-four hours post transfection, cells were processed for total RNA isolation, qRT-PCR and RNA-seq. Not surprisingly, while some viral RNA knockdown was observed in all conditions, the greatest reduction was observed with the enzymatically active Cas13a and guide PB2_g1 (Fig 4A and 4B). The normalized coverage along the PB2 segment was decreased only in the Cas13a+PB2_g1 guide treatment (Fig 4C and S10 Table). However, between positions 2170 and 2280, decrease in coverage was observed in all conditions, and more dramatically so in the Cas13a+PB2_g1 guide treatment, consistent with the qPCR results (Fig 4D and S10 Table). Consequently, cleavage or degradation by Cas13 is not uniform across the PB2 genomic segment but it is augmented in close proximity to Cas13’s binding site, within the conserved sequence. Critically, Cas13’s enzymatic activity was needed to induce the KD of other viral segments (Fig 4E and 4F, and S10 Table). Accordingly, treatment with Cas13 induced ~90% KD in cells infected at higher MOIs, while the guide alone lost potency (S7A–S7D Fig).

These results indicate that binding of the Cas13/guide complex or the guide alone, exemplified by the dCas13a and GFP conditions, respectively, may decrease RNA levels, and they are likely part of the mechanism through which Cas13’s mediated RNA KD is achieved. However, Cas13’s enzymatic activity is needed to induce degradation of other (non-PB2) viral RNA segments and induce potent mitigation of the infection.

### Guide PB2_g1 induces efficient viral KD across H1N1 and H3N2 influenza stains

**Fig 5:**
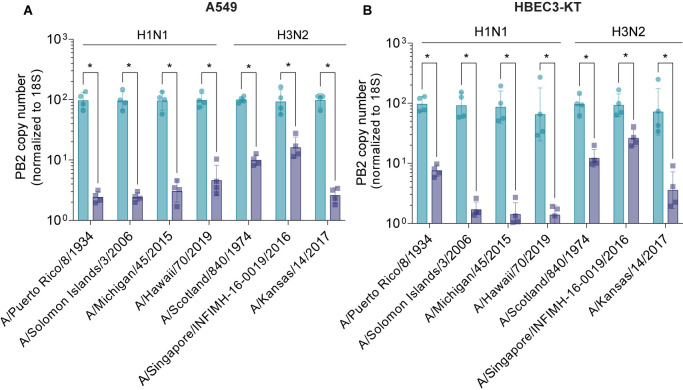
Efficacy of guide PB2_g1 across H1N1 and H3N2 Influenza A strains. (A) Fold change of PB2 RNA levels normalized to the NTCR condition in A549 cells infected with the indicated Influenza A strains and treated with Cas13 mRNA and guide PB2_g1 guide using the primer/probe set in S2 Table. *p 0.000005 (Multiple unpaired t test comparisons on log-transformed data). (B) Fold change of PB2 RNA levels normalized to the NTCR condition in HBEC3-KT cells infected with the indicated Influenza A strains and treated with Cas13 mRNA and guide PB2_g1 guide using the primer/probe set in S2 Table. *p 0.000008 (Multiple unpaired t test comparisons on log-transformed data). The bars represent mean ± s.d. n = 4 per condition.

Since guide PB2_g1 was designed to target a conserved region across > 50,000 influenza A strains, we next tested its efficacy *in vitro* against a panel of recently circulating H1N1 and H3N2 viruses. The experiment was initially performed in A549 cells, as described above. After infection and treatment, total RNA was extracted and analyzed by qRT-PCR using a primer-probe set designed across the Cas13 binding site (S2 Table). Guide PB2_g1 efficiently decreased viral RNA in all experimental conditions, with significant differences in potency across H1N1 and H3N2 strains (Fig 5A). This was likely due to isolate specific differences in infectivity, because no mismatches were observed in the target sequences (S11 Table). The results were confirmed in HBEC3-KT cells, an hTERT-immortalized bronchial epithelial primary cell line used in toxicology research (Fig 5B). Last, guide PB2_g1 demonstrated strong and sustained KD of PB2 across MOIs of A/H1N1/Puerto Rico/8/1934, A/H1N1/California/07/09, A/H3N2/Hong Kong/267/2019 and A/H1N1/WSN/1933 (S8 Fig). On the contrary, guide PB2_m4 efficiently KD PB2 of both A/ H1N1/Puerto Rico/8/1934 and A/H1N1/WSN/1933 in cells infected with low MOI, but its potency was significantly reduced at higher MOIs, consistent with our previous results (S9 Fig). Together, these findings indicate that targeting influenza A viral genome with guide PB2_g1 provides consistent and potent mitigation of infection across several IAV strains and MOIs.

### Screening of PB2_g1 guides with chemical modifications

**Fig 6:**
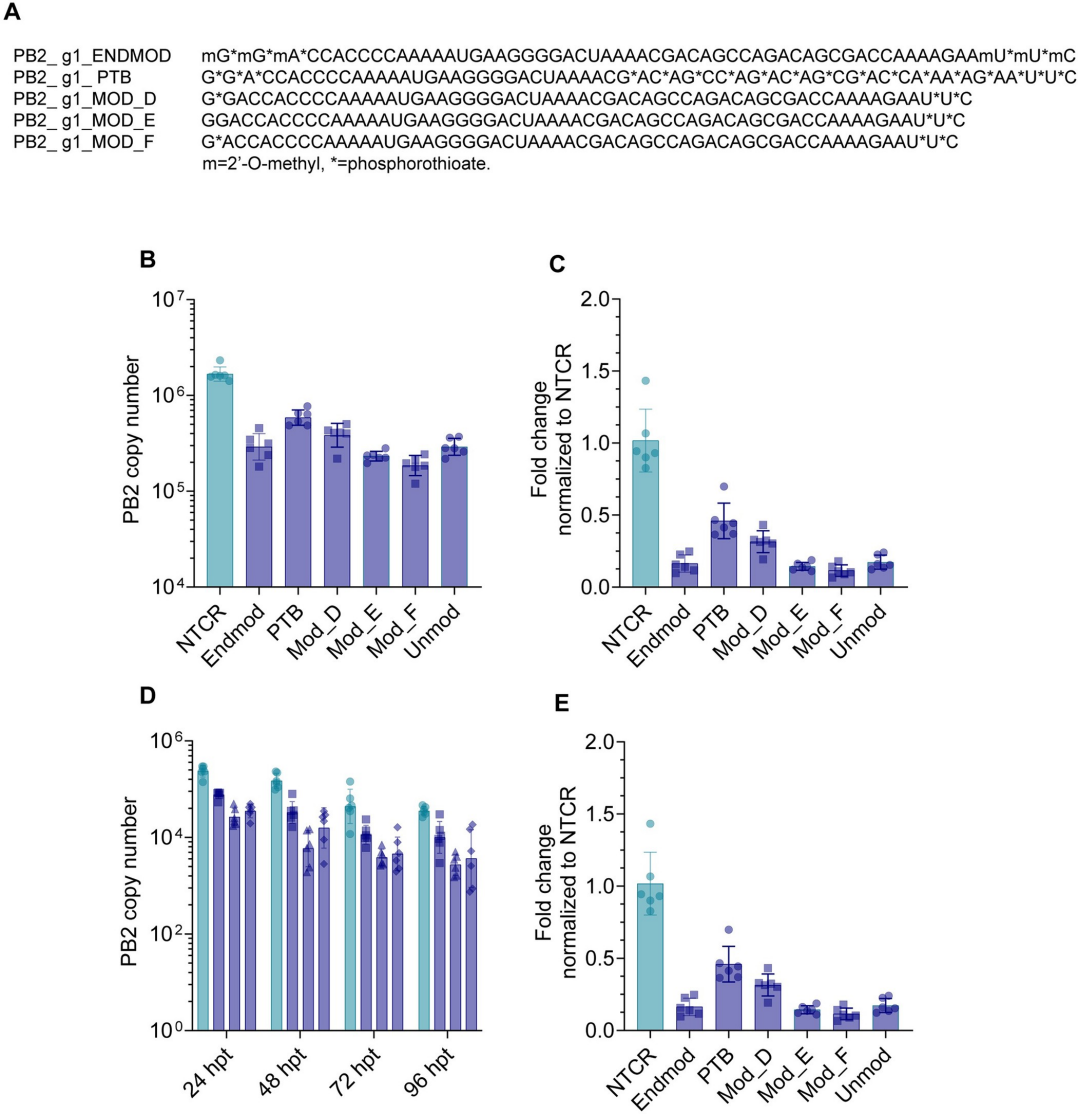
Screening of PB2_g1 chemically modified guides. (A) Details of the chemical modifications designed for guide PB2_g1. **(**B) Copy number of PB2 RNA in A549 cells infected with A/H1N1/California/04/09 at MOI 0.01 and treated with Cas13 mRNA and PB2_g1 genome targeting guides with the indicated chemical modifications using primer/probe Set 5. (C) Fold change of PB2 RNA levels normalized to the NTCR condition for the data in part B. (D) Copy number of PB2 RNA in A549 cells infected with A/H1N1/California/04/09 at MOI 0.01 and treated with Cas13 mRNA and PB2_g1 genome targeting guides with the indicated chemical modifications using primer/probe Set 5. Cells were processed for qPCR at the indicated hours post-transfection. (E) Fold change of PB2 RNA levels normalized to the NTCR condition for the data in part D. In all parts bars represent mean ± s.d. n = 6 per condition. *p*-values for each comparison are listed in S12 Table.

In preparation for *in vivo* challenge experiments, we screened PB2_g1 guides with different chemical modifications to improve their RNA targeting efficiency and increase their half-life [26]. We designed six guides with 2’O-methyl and/or phosphorothioate modifications either at each end of the guide’s sequence or within the target sequence (Fig 6A and S1 Table). The guides were then tested in A549 cells, as described above, and their effect was analyzed by qPCR. Among all modified guides, guide PB2_g1 Mod_F displayed the strongest level of PB2 KD (~ 90%, Fig 6B and 6C, and S12 Table). To evaluate if PB2 knockdown would persist over time, we repeated the assay, and processed cells for qPCR at 24, 48, 72 and 96 hpt, as a lasting antiviral effect is required to avoid virus rebound. Guide PB2_g1 containing modification Mod_F maintained the strongest level of PB2 KD over 96h (Fig 6D and 6E, and S12 Table) and it was therefore selected for the *in vivo* challenge.

### Nebulized mRNA-encoded Cas13 and guide RNA PB1_g1 mitigate influenza infection in hamsters

Once we demonstrated that Cas13 and guide PB2_g1 reduce IAV infection *in vitro*, we sought to evaluate their efficacy in golden syrian hamster, an animal model previously used for influenza and SARS-CoV-2 mitigation studies [27]. To characterize infection by the intranasal route of administration, hamsters were infected with 10^5^ PFU of A/H1N1/California/04/09, lungs were collected at 2, 3, 4, 5 and 7 days post-infection (DPI) and processed for PB2 viral RNA quantification by qPCR and viral titer by plaque assay. The highest levels of RNA copy number and viral titer were observed at 2 dpi (S10A–S10C Fig), thus establishing it as endpoint for all challenge experiments. Animal weights were not significantly affected by the infection (S13 Table).

Then, we optimized delivery of the RNA cargos via nebulization to hamster lungs, in order to determine the appropriate dose to be administered for each animal. An mRNA encoding for LbuCas13a fused to a nanoluciferase (Cas13-NLuc) reporter was formulated, along with guide PB2_g1, with the poly- β- amino thio-ester (PBATE) P76, which demonstrated potent delivery to several animal species and mitigation of SARS-CoV-2 in hamster [24,25]. The formulated RNA was concentrated, to increase the effective dose delivered, and it was administered to hamsters using the previously described FDA approved nebulizer and custom dosing apparatus. RNA doses of 200, 300, and 400 μg per animal were added dropwise to ensure maximum nebulization and delivery of payload. Luminescence of the hamsters’ lungs was measured by IVIS 24h post-delivery, supporting a dose of 300 μg of mRNA per animal (S10D Fig). To confirm efficacy, mRNA encoding for LbuCas13a was delivered to the lung of hamster along with a guide targeting an endogenous gene, TMEM41B, and KD was assessed 48h post-delivery by qPCR, revealing ~60% reduction of the transcript (S10E Fig).

**Fig 7:**
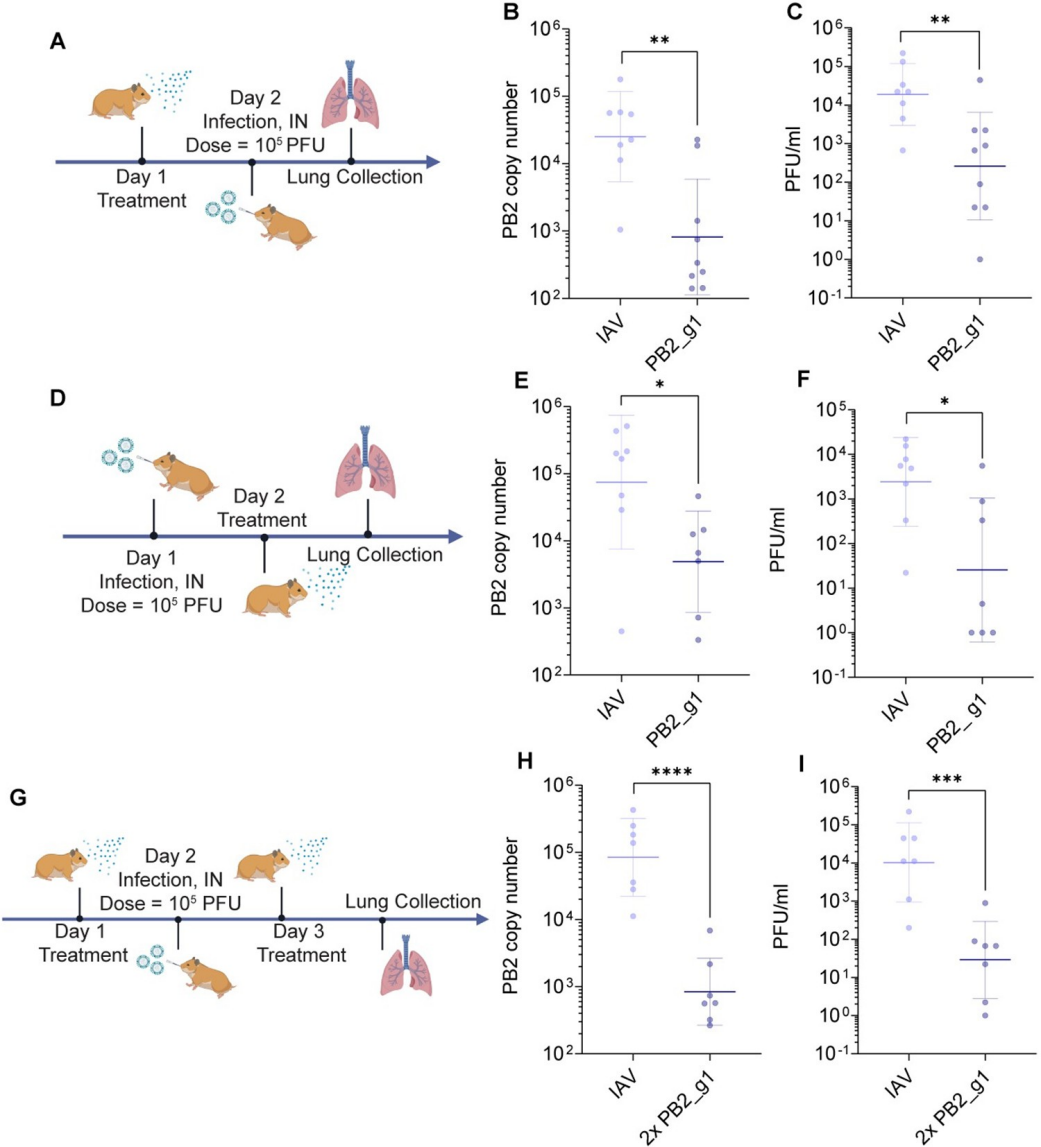
Nebulization of mRNA-expressed Cas13a and guide PB2_g1 against Influenza infection. (A) Schematic representation of the *in vivo* experimental design for mitigation of A/H1N1/California/04/09 infection after nebulization of Cas13a and guide PB2_g1 formulated with polymer P76 and delivered prophylactically. (B) Copy number of PB2 RNA in lungs of hamsters infected with A/H1N1/California/04/09 10^5^ PFU and untreated (Cal/04/09) or treated 24h before infection with Cas13 mRNA and guide PB2_g1. The bars represent mean ± s.d. n = 8 per condition. **p 0.0013 (Unpaired t test comparison on log-transformed data). (C) Lung viral titers for experiments in part A. The bars represent mean ± s.d. n = 8 per condition. **p 0.0047 (Unpaired t test comparison on log-transformed data). (D) Schematic representation of the *in vivo* experimental design for mitigation of A/H1N1/California/04/09 infection after nebulization of Cas13a and guide PB2_g1 formulated with polymer P76 and delivered as treatment 24h post infection. (E) Copy number of PB2 RNA in lungs of hamsters infected with A/H1N1/California/04/09 10^5^ PFU and untreated (Virus only) or treated 24h before infection with Cas13 mRNA and guide PB2_g1. The bars represent mean ± s.d. n = 8 per condition. **p 0.0236 (Unpaired t test comparison on log-transformed data). (F) Lung viral titers for experiments in part E. The bars represent mean ± s.d. n = 8 per condition. **p 0.0125 (Unpaired t test comparison on log-transformed data). (G) Schematic representation of the *in vivo* experimental design for mitigation of A/H1N1/California/04/09 infection after nebulization of Cas13a and guide PB2_g1 formulated with polymer P76 and delivered before and after infection. (H) Copy number of PB2 RNA in lungs of hamsters infected with A/H1N1/California/04/09 10^5^ PFU and untreated (Virus only) or treated before and after infection with Cas13 mRNA and guide PB2_g1. The bars represent mean ± s.d. n = 7 per condition. ****p<0.0001 (Unpaired t test comparison on log-transformed data). (I) Lung viral titers for experiments in part H. The bars represent mean ± s.d. n = 7 per condition. **p 0.0006 (Unpaired t test comparison on log-transformed data). Fig 7A and 7D and 7G were created in part using BioRender.com.

Last, we evaluated efficacy against influenza with different treatment regimens. Hamsters were first dosed with Cas13 and guide PB2_g1. 24h later, animals were inoculated intranasally with 10^5^ PFU of influenza A/H1N1/California/04/09. Animals’ lungs were isolated 48 hr post-infection and analyzed by qPCR to quantify PB2 RNA copy numbers and infectious viral titer by plaque assay (Fig 7A). Following prophylactic treatment with a single dose, we observed ~1-log reduction in RNA and infectious virus (PFU/ml) in the lungs of treated animals compared to IAV infected untreated controls (Fig 7B and 7C). We next evaluated Cas13 and guide PB2_g1 efficacy when delivered as treatment, after IAV infection (Fig 7D). Forty-eight hours post infection hamster lungs were isolated and qPCR and plaque assay were performed. Treatment with Cas13 resulted in 93% KD of PB2 copy number and 88% reduction in viral titer, demonstrating efficacy as a treatment (Fig 7E and 7F). Last, we assessed the effect of a two-dose regimen, in comparison to the prophylactic and therapeutic treatments, by delivering to hamsters a Cas13 and guide PB2_g1 before and after infection (Fig 7G). This double dose regimen induced ~2-logs reduction in both RNA and infectious virus (PFU/ml) in the lungs (Fig 7H and 7I). Animal weights were not significantly affected by any treatment (S14 Table). Of note, qPCR results were reported both as PB2 copy numbers and as PB2 fold change using 18S as an endogenous control, as its expression was not significantly affected by the treatment, with the exception of a slight 2.3-fold change in the double dose experiment (S11 Fig and S15 Table).

Taken together, these results demonstrate that both therapeutic and a combination prophylactic and therapeutic treatment regimen with nebulized mRNA-expressed Cas13a and guide PB2_g1 RNA leads to significant mitigation of IAV infection. A two-dose regimen strengthens the effectiveness of the treatment by further reducing RNA and viral titer.

## Discussion

The CRISPR-Cas13 system represents a very attractive alternative therapeutics platform against RNA viruses, as demonstrated by several publications where significant viral KD was observed upon delivery or overexpression of different Cas13 effectors against SARS-CoV-2 [28–31], Dengue [32,33], Zika [34], PRRSV [35], BDV [36] and human enterovirus [37] in different cell lines and *in vivo*. However, much remains to be elucidated to further develop Cas13 as an antiviral, especially in light of recent publications reporting Cas13 mediated toxicity and collateral RNA cleavage in eukaryotic cells, hinting to significant safety issues [38–42]. In our previous and current work, LbuCas13a was expressed via the mRNA platform, and guides were produced via solid-state synthesis. This is widely recognized as a safer approach because mRNA expression is transient and limited to the cell cytosol. Moreover, the mRNA is chemically modified to reduce host innate immune responses, while boosting the expression of the encoded protein. This approach is also programable: we can express a cytosolic version of Cas13 or a nuclear version or both, depending on the viral target and its lifecycle. Different RNA viruses can be targeted just by changing the sequence of the guide.

Here, a series of *in vitro* assays were designed and performed toward the identification of a strong candidate guide with potent KD of A/H1N1/California/04/09. In all assays performed, A549 cells were first infected with the virus for 24h, followed by co-transfection of mRNA-encoded LbuCas13a+LbuCas13-NLS and a guide for 24h, before processing for efficacy. We re-evaluated the potency of one mRNA (PB2_m4) and one genome (PB2_g1) targeting guide and showed that in our previous work we underestimated the efficiency of viral RNA knockdown by the genome targeting guide. This was demonstrated by performing RT-qPCR analysis at multiple sites along the target RNAs, and the results were confirmed by RNA-Seq. Indeed, while targeting PB2 mRNA was effective, targeting the PB2 genomic sequence provided a more potent mitigation of infection as demonstrated by lower viral copy number across all segments and viral titer, and sustained efficacy across multiplicities of infection. Interestingly, guide PB2_g1 targets the PSL2 stem-loop structure, which serves as a packaging signal for the genome segment [43]. Disruption of this motif by antisense oligonucleotides (ASOs) was shown to dramatically inhibit IAV infection of various strains and subtypes, with limited development of resistance, unlike Oseltamivir, *in vitro*. Accordingly, treatment with Cas13 and guide PB2_g1 induced significant KD of various H1N1 and H3N2 influenza strains *in vitro*, and the RNase activity was needed for sustained viral KD. Delivery of the guide alone (or of an enzymatically defective Cas13) resulted in reduced or negligible KD of PB2 and other segments and reduced mitigation of influenza infection at high MOI.

The RNA-Seq data revealed strong correlation with the qRT-PCR results, suggesting that the latter is a suitable approach to evaluate Cas13a viral knockdown. It should be noted that we routinely reported our qPCR data both as absolute PB2 copy number or as fold change normalized to GAPDH or 18S, as endogenous controls. Throughout our experiments, we did not observe significant changes in the expression of either, *in vitro* or *in vivo*. These results are also consistent with previous data where no off-target effects were observed when Cas13 was used to KD the endogenous genes KRAS, CXCR4 and PPIB. These results are instead in sharp contrast with recent publications, where collateral cleavage was observed when using several Cas13 RNAses in eukaryotic cells, resulting in degradation of endogenous transcripts, upregulated stress and innate immune response genes and, in certain conditions, apoptotic cell death [39]. The authors acknowledged that this phenomenon may depend on the methods used to deliver either Cas13 and/or the guides, it is particularly evident when the transcript to be KD is a reporter mRNA overexpressed by the cell, it appears to be cell-type dependent and overall may be impacted by the lack of rigorous controls to account for all conditions. Using the mRNA platform, no cell death was observed.

After establishing guide PB2_g1 as the best candidate for potent mitigation of Cal04 in A549 cells, guides with different chemical modifications at the ends of the guides, as well as within the spacer sequence, were screened, to improve RNA targeting efficiency and improve crRNA stability [26]. Among the guides screened, PB2_g1 with MOD_F induced potent PB2 KD, which persisted over 96h. This guide contains only 4 phosphorothioate modified bases: one at the 5’ end G and 3 at the 3’ end TTC. In addition, one of the 5’end Gs was removed, to prevent formation of G-C interaction within the direct repeat sequence or between the direct repeat and the spacer sequence. The most heavily modified guide, MOD_B, was the least potent. This result corroborates previous data indicating that addition of modified bases within the crRNA must be carefully evaluated, likely on a guide-to-guide basis, to avoid disruption of Cas13-crRNA interactions.

Last, the efficacy of Cas13 and guides against influenza was assessed in the hamster model of infection. Hamsters were chosen for two main reasons: first, because they have been previously used in similar studies with both IAV and IBV strains, as well as in transmission and coinfection studies with SARS-CoV-2 [23,44,45]. Second, we demonstrated efficacy of mRNA-expressed Cas13 against SARS-CoV-2 in this animal model, upon formulation with a PBATE polymer-based delivery vehicle (P76) and delivery to the lungs by nebulization [22,24]. Concentrating the polymeric formulation to increase the dose of delivered mRNA and guide to 300 μg /animal, allowed for increased expression, without significant toxicity [25]. Prophylactic treatment with a single dose resulted in ~1-log reduction in viral RNA and titer, while delivery as a treatment, 24h post infection, resulted in 93% KD of PB2 copy number and 88% reduction in viral titer. It is clear that there is a KD threshold of viral RNA that needs to be reached to observe significant reduction of viral titer and achieve therapeutic levels. As a confirmation, administration of a double dose of Cas13 and guide, before and after infection, induced ~2-logs reduction in both RNA and infectious virus (PFU/ml) in the lungs.

In conclusion, we described an experimental workflow that allowed to identify a Cas13 crRNA with potent KD of PB2, leading to substantial mitigation of IAV infection *in vitro* and *in vivo*, without significant off target effects and toxicity. Using the hamster model of infection, the platform will be expanded to additional studies aimed to mitigate infections by IBV and SARS-CoV-2, toward the development of a pan-respiratory drug.

## Methods

### Ethics statement

Animal studies were performed under ethical guidance and upon approval from the University of Georgia Animal Health Research Center and the Georgia Institute of Technology Institutional Animal Care and Use Committee (IACUC) following National Institutes of Health (NIH) guidelines under Protocols numbers AUP 2023 02-013 and A100169, respectively.

### Cell lines and viruses

All the cell lines used in this work were purchased from American Type Culture Collection (ATCC). Human lung epithelial cells A549 (CCL185), MDCK cells (Madin-Darby canine kidney) and HBEC3-KT cells were propagated according to ATCC recommendations. Influenza virus stocks (H1N1 –A/H1N1/WSN/33, A/H1N1/California/04/09, A/H1N1/Puerto Rico/8/1934, A/H1N1/California/07/09 and A/H3N2/Hong Kong/26/2019) were grown in MDCK cells. Briefly, MDCK cells were grown until 90% confluence in 175 mm^2^ flasks. The next day, cells were washed once with PBS, and infected with low volume of serum-free media (1X MEM) containing virus (MOI 0.001) for 1 hour at room temperature (RT). Then, infection media (1XMEM + 4% v/v BSA + TPCK Trypsin 1:1000 dilution—Worthington Biochemical Corporation) was added to a final volume of 15 ml. Cells were monitored for 72 hours until CPE was observed. The supernatant was collected and centrifuged at 1,000 x g for 10 minutes. Virus samples were aliquoted and stored at -80°C for future use. Influenza virus strains used in Fig 5 were obtained from BEI Resources, the International Reagent Resource, the American Type Culture Collection (ATCC), or were kindly provided by Dr. Peter Palese. Viruses were grown on MDCK cells or in embryonated chicken eggs at 37°C for 48–72 hours. To verify the identity of each strain, viral RNA was isolated using TRIzol reagent (Invitrogen 15596026) and segments 4, 6, and either 3 or 5 were amplified using the SuperScript III One-Step RT-PCR System (Invitrogen 12574–035) followed by Sanger sequencing and comparison to reference sequences. Reference sequences for comparison were unable to be located for A/Scotland/840/1974; the closest matching reference sequences however, were A/Albany/20/1974 for segment 4 (CY021093.1), segment 5 (CY021096.1), and segment 6 (CY021095.1).

### Plaque assay *in vitro*

Virus titer was determined by immunostaining plaque assay. MDCK cells were plated at 400,000 cells per well 24 hours prior titration. Samples were diluted 10-fold up to 10^−11^ in 1XMEM + TPCK Trypsin (1μg/ml). Cells were washed twice with PBS and diluted samples were inoculated in triplicate. Cells were incubated at 37°C, rocking the plates every 15 mins to allow for virus adsorption. After 60 min, 2ml of overlay media was added to each well, consisting of 1 ml of Avicell 3.2% and 1 ml of 2X media (2X MEM, 40 mM Hepes Solution, 4 mM L-glutamine, 0.15% NaCHO_3_ and 2% Pen/Strep/Amp B Solution). Samples were incubated for 72 hours at 37°C. After incubation, media was removed, and cells were washed once with PBS and fixed with 4% PFA for 10 mins at RT. Cells were washed once with PBS and blocked with 5% BSA for 30 mins at 37°C. After blocking, cells were washed once with PBS and incubated with pan-influenza primary antibody (1:1000 dilution, Abcam #ab20841) for 30 mins at 37°C. Cells were washed once with PBS and incubated with secondary HRP antibody (1:500 dilution, Jackson ImmunoResearch Laboratories #705-035-003) at 37°C for 30 min. Then, cells were washed once with PBS and incubated with TrueBlue substrate for 10 min at RT. Cells were washed once with water and titer was determined according to the formula PFU/ml = average number of plaques x (1/dilution factor) x (1000/inoculum volume).

### mRNA encoded-Cas13 constructs

All the mRNA used in this work were designed and synthesized by in vitro transcription (IVT) reaction as described in Blanchard et al (Lbu Cas13a, LbuCas13-NLS, dLbuCas13a and GFP) and in Rotolo et al (LbuCas13a-NLuc) [22,24]. Briefly, plasmids (GenScript) were linearized with NotI-HF (NEB) overnight at 37°C, purified by sodium acetate (Thermo Fisher Scientific) precipitation and rehydrated with nuclease-free water. *In vitro* transcription was performed overnight at 37°C using the HiScribe T7 kit (NEB) following the manufacturer’s instructions (N1-methyl-pseudouridine modified). The resulting RNA was treated with DNase I (Aldevron) for 30 min to remove the template, and it was purified using lithium chloride precipitation (Thermo Fisher Scientific). The RNA was heat denatured at 65°C for 10 min before capping with a Cap-1 structure using guanylyltransferase and 2’-O-methyltransferase (Aldevron). mRNA was then purified by lithium chloride precipitation, treated with alkaline phosphatase (NEB) and purified again. The mRNA concentration was measured using a NanoDrop instrument. Purified mRNA products were analysed by gel electrophoresis to ensure purity. All unmodified guides (crRNA) used in this work were purchased from Synthego and all modified guides were purchased from IDT (S1 Table).

### Cell-free detection assay

LbuCas13a protein was first synthesized from in vitro transcribed mRNA through in vitro translation using a Nuclease-Treated Rabbit Reticulocyte Lysate (Promega), according to manufacturer’s instructions.

A reaction mixture was prepared by combining each crRNA (final concentration 2.5 nM), synthetic target RNA (final concentration 47.5 nM), and poly-U10 fluorescent reporter (final concentration 1 μM) in RNA Processing Buffer composed of 20 mM HEPES (pH 6.8), 50 mM KCl, 5 mM MgCl2, BSA (10 μg mL-1), yeast tRNA (10 μg mL-1), TCEP (1 μM) and 5% glycerol. The reaction mixture was added to a 96-well plate and combined with 10% by volume of the in vitro translated protein. Fluorescence excitation at 485 nm was measured at an emission wavelength of 528 nm for 90 minutes in 10-minute intervals.

### In vitro antiviral assays against influenza

A549 cells were seeded overnight at a density of 130,000–135,000 cells per well in a 24-well plate. The next day, cells were washed once with PBS and infected with influenza virus (different MOIs) diluted in serum-free 1X MEM media + 1μg/ml TPCK Trypsin for 1 hour. After, the inoculum was removed, infection media was added (1XMEM + 4% v/v BSA + 1μg/ml TPCK Trypsin) for 24 hours. Cells were then transfected with mRNA encoding LbuCas13a (with and without NLS 250 ng each), dLbuCas13a (500ng) or GFP (200 ng) and 50x molar excess targeted crRNA or non-targeted crRNA guides using Lipofectamine Messenger Max (Thermo Fisher Scientific) according to manufacturer instructions and as described^19^. The sequences of the crRNA guides are detailed in S1 Table. Twenty-four hours post-transfection (hpt) total RNA was extracted using RNeasy Plus Mini Kit (Qiagen) and quantified by Nanodrop. cDNA was prepared using High-Capacity cDNA Reverse Transcription Kit (Applied Biosystems, Thermo Fisher Scientific). qPCR experiments were performed using the FastAdvanced Master Mix (Thermo Fisher Scientific). The antiviral activity of Cas13a system was measured by qPCR with absolute quantification of the viral PB2 gene copy number and fold change with respect to either GAPDH or 18S as endogenous controls. Experiments were performed using QuantStudio7 Flex thermal cycler (Applied Biosystems).

For the experiments described in Fig 5, A549 cells were infected with viruses at MOI 0.1 except PR8 (MOI 0.01), Michigan (MOI 0.5), and Hawaii (MOI 0.5). HBEC3-KT cells were infected with viruses at MOI 0.1 except PR8 (MOI 0.01), Michigan (MOI 0.5), and Hawaii (MOI 1). A549 or HBEC3-KT cells were seeded at a density of 90,000 cells/well in a 24-well plate. The next day, cells were infected with virus diluted in PBS/BSA infection media (PBS, 0.4% BSA, Mg^2^/Ca^2+^, penicillin/streptomycin) for 1 hour at 37°C. Virus was then removed and replaced with post-infection media (OptiMEM, 0.35% BSA, 0.01% FBS, penicillin/streptomycin) and 0.3 μg/mL TPCK/trypsin. Right before transfections, post-infection media was replaced with fresh post-infection media and TPCK/trypsin. A549 and HBEC3-KT cell lines were transfected at 24hpi with 250ng each Cas13a and Cas13a (NLS) mRNA and 360ng guide mRNA using Lipofectamine MessengerMAX (Thermo Fisher). RNA samples were collected from tissue cultures using the Monarch Total RNA Miniprep Kit (New England BioLabs) 24 hours post transfection. Prepped RNA samples were then analyzed using the EXPRESS Superscript One-Step qRT-PCR kit (Thermo Fisher) with eukaryotic 18S rRNA (Applied Biosystems) and primers/probe targeting PB2 (IDT) on an Applied Biosystems QuantStudio 3 instrument.

### Next-generation sequencing

Total RNA from each experimental condition described in Figs 1 and 4 was extracted as described above. 2μg of purified total RNA was sent to Azenta for rRNA depletion, cDNA library preparation, and Illumina RNA sequencing with approximately 30M paired-end reads per sample. For the data in Fig 1, reads were filtered, trimmed, and aligned to the A/H1N1/California/04/09 gene segment sequences (REF FJ966079, REF FJ966080, REF FJ966081, REF FJ966082, REF FJ966083, REF FJ966084, REF FJ966085 and REF FJ966086) using the Geneious RNA algorithm (Geneious Prime 2020.4). Coverage at each nucleotide position for each influenza genome segment was exported against the consensus sequence without gaps. Normalized coverage was calculated by dividing the coverage at each position by the average coverage of the virus only samples at that position. Viral RNA counts of each segment were determined counting ambiguously mapped reads as partial matches. For the data in Fig 4 quality checks were performed before and after adapter trimming using FastQC (v0.11.8) and fastq-screen (v0.15.3) to assess for read quality, adapter content, and reference species confirmation [46]. Trimmomatic v0.36 was used to remove trim Illumina adapters before downstream processing [47]. MultiQC v1.19 was applied to the output of FastQC and fastq-screen to yield confirmation of read quality and successful trimming [48]. Detection of Influenza A in the samples also used the trimmed FASTQ files as input. The viral reference genome and segment annotation for the influenza virus was obtained through NCBI using accession numbers MK159426, MK159425, MK159424, MK159423, MK159422, MK15942,1MK159420, and MK159419 for H1N1 (influenza A virus [A/H1N1/California/04/2009]). The segment annotation GFF file was converted to a GTF for input to the STAR v2.7.1a alignment tool using cufflinks [49,50]. Indexing of the H1N1 reference genome was performed using STAR with parameter—genomeSAindexNbases set to 5 due to the very small length of the genome (as recommended by the developer, for when log_2_(genome_length)/2–1 is less than the SA index argument default, which is 14). Alignment to the reference was performed using STAR with parameters—quantMode GeneCounts,—outFilterMismatchNoverReadLmax 0.03, and—chimSegmentMin 20 [49]. Samtools v1.14 was used to sort the alignment files, while read counts and analytical information were obtained using samtools idxstats, samtools flagstat, and MultiQC v1.19 [48,51].

### Cell viability assay

A549 cells were seeded overnight at a density of 35,000 cells per well in a 96-well plate and then infected and/or transfected in triplicates per condition as described above. Twenty-four hours post transfection cells were processed using a live/dead viability/cytotoxicity kit for mammalian cells (L3224, Molecular Probes) following manufacturer’s instructions for 30 minutes before measuring the fluorescence signal. A set of cells were incubated with 70% methanol for 30 minutes before treatment to assess the fluorescence signal associated to dead cells. The percentage of live and dead cells was quantified by measuring the fluorescence signal at 494/517 nm for Calcein AM and at 528/617 nm for Ethidium Homodimer-1 on a Synergy H1 plate reader (Biotek) following manufacturer’s instruction.

### Animal studies

Hamster delivery optimization experiments were performed at the Georgia Institute of Technology. Four-week-old male LVG golden Syrian hamsters (Charles River Laboratories) were maintained in individually ventilated and watered cages kept at negative pressure. The hamsters were kept in rooms on a 12 h light/dark cycle with ambient temperature between 21.1 and 22.8°C with 35–50% relative humidity. The experiments were only performed during the light phase. Food (Lab Diet 5001) was provided to hamsters ad libitum. The animals were acclimatized for at least 6 days before beginning the experiments. The animals were randomly distributed among the experimental groups. Researchers were blinded to the animal group allocation during data acquisition. The animals were euthanized by CO2 asphyxiation. Assessment of *in vivo* efficacy of Cas13a mRNA and crRNA was performed at the University of Georgia Animal Health Research (AHRC) facility. Outbred male LVG golden Syrian hamsters (Charles River Laboratories) were received at 24–47 days of age weighing 65–85 g. The animals were randomly distributed among experimental groups by animal care staff blinded as to study design and treatment. The animals were housed in individually ventilated and watered cages kept at negative pressure (n = 4 animals per cage) in rooms with a 12 h light/dark cycle, an ambient temperature between 21.1 and 22.8°C and 35–50% relative humidity. Food (Lab Diet 5001) was provided ad libitum. Upon receipt, hamsters were allowed to acclimate for at least 6 days before experiments were initiated. Infected animals were handled and kept under BSL-2 conditions until euthanized.

### Influenza infections in hamster

Hamsters were anesthetized by intraperitoneal injection with a combination of 100 mg/ kg of ketamine and 5 mg/kg of xylazine. After loss of toe pinch reflex, 10^5^ PFU of influenza A/H1N1/California/04/09 in 50 μl 1x PBS was administered dropwise to each animal via intranasal route. Animals were then administered reversal agent (atipamezole, 0.15 mg/kg) and place on a heating pad until they were able to right themselves. Body weights and clinical signs were checked and recorded daily. Animals were sacrificed at the indicated timepoints when lungs were frozen on dry ice and kept at −80°C until all time points were complete. Last, lungs were processed for qPCR and plaque assay as described below.

### Nebulizer-based RNA delivery to hamsters

PBATE P76 was synthesized as previously described [24] with the following differences: 3 equivalents of 2-methylpentane-1,5-diamine were used to avoid incomplete end-capping and cross-coupling between the polymer chains. The polymer was purified thrice by reprecipitation from methanol into diethyl ether to obtain a white powder. Before delivery to animals, 100 Mm of sodium acetate pH 5.0 was used to solubilize both the PBATE P76 at 25 mg/mL and mRNA at 1 mg/mL before mixing. The final concentration of the mRNA was 0.5 mg/ml, and the PBATE was used at a 25× mass ratio to the mRNA. The tubes were vortexed briefly to mix and incubated at room temperature for 10 min. To concentrate, polyplexes were filtered using a 30 kDa cut-off centrifugal filter (Amicon Ultra) for 30min at 4°C at 2000 × g. Filters were then inverted into a new tube, and retentate was collected by centrifugation for 5 min at 4°C at 1000 × g. The volume of the retentate was then measured and used to calculate the volume of delivery needed to achieve the indicated doses. Hamsters were placed into restraint tubes (CODA Large Mouse Holder, Kent Scientific) fitted with a custom 3D-printed nose cone (3D Printing Tech) as described before. These were then inserted into an exposure system constructed of a clear PVC tee fitted with an Aerogen Aeroneb Solo nebulizer (Tri-anim, 06-AG-AS3200) placed in the upward facing port of the tee (n = 2 restraints per setup). Doses were added dropwise.

### Optimization of delivery by Luminescence *in vivo*

The indicated doses (200, 300 and 400 μg) of LbuCas13a-NLuc and guide PB2_g1 at a 60:40 mass ratio of were formulated with P76 polymer at 1:25 ratio and were delivered dropwise as previously described. After 24h hamsters were euthanized, whole lungs were collected and rinsed with PBS. Lungs were then placed into a solution of Nano-Glo substrate solution (Promega) diluted 50-fold in PBS. Lungs were incubated for 5 min and then placed onto black paper and imaged with an IVIS Spectrum CT (PerkinElmer). Lung luminescence was then quantified using Living Image software (version 4.7.4, PerkinElmer)

### TMEM41B KD *in vivo*

Hamsters were treated with 300 μg of LbuCas13a and TMEM41b targeting guide at a 60:40 mass ratio formulated with P76 polymer at 1:25 ratio as previously described. After 24h hamsters were euthanized, whole lungs were collected in Trizol, homogenized and processed for qPCR as described below. The fold change of TMEM41b expression was measured using GAPDH as endogenous control.

### Challenge against influenza *in vivo*

Briefly, for all experiments 300 μg at a 60:40 mass ratio of Cas13a mRNA to PB2_g1 guide RNA formulated with P76 polymer at 1:25 ratio was delivered dropwise per hamster at each administration as previously described. For two dose experiments (pre- and post-infection combination), a total of 184 μl (92 μl x2) was delivered as the pre-infection dose and a total of 350 μl (116 μl drops x3) was delivered as the post-infection dose. For post-infection treatment experiments, a total of 350 μl (116 μl drops x3) was delivered. Of note, although volumes administered differ the mass of RNA delivered was equal. After each droplet was nebulized, the clear tee was inspected until the vaporized dose had cleared (approximately 15–45 s per drop). After the vapor had cleared following to the last droplet, the hamsters were removed from restraints and placed back into cages. Animals were sacrificed 24 hr after the final treatment.

### qPCR assay *in vivo*

After euthanasia, whole lungs were collected in C tubes (Miltenyi) containing 2 ml MEM 1x antibiotic-antimycotic solution (Gibco) and dissociated using a GentleMACS homogenizer using the “lung-2” program (Miltenyi). Homogenates were centrifuged at 3000 RPM for 10 min at 4°C. The supernatant was removed, aliquoted and stored at -80°C. One aliquot of supernatant (100 μl) was added to 900 μl Trizol and chloroform phase separation was performed. The aqueous fraction was combined with 70% ethanol and RNA was then extracted using conventional centrifugal columns (RNeasy Plus Mini Kit, Qiagen). Total RNA was quantified using a Nanodrop and 0.5–1 μg was used for cDNA synthesis using the SuperScript VILO cDNA Synthesis Kit after DNase I treatment with eZDNAse according to manufacturer’s instructions (Thermo Fisher Scientific).

qPCR experiments were performed using the FastAdvanced Master Mix (Thermo Fisher Scientific). Copy number was determined using a DNA oligo standard (IDT) and fold change was calculated using 18S as endogenous control. Experiments were performed using a QuantStudio7 Flex thermal cycler (Applied Biosystems).

### Plaque assay *in vivo*

Viral titer in the lungs was determined by plaque assay in MDCK cells. Briefly, supernatants from whole lung homogenates were thawed on ice and serially diluted 10-fold in MEM with 1 μg/ml TPCK-trypsin. MDCK cells were seeded into 12-well plates (Corning Costar, Cambridge, MA) and allowed to grow over night to 90% confluency. Dilutions (450 μl) were inoculated in duplicate onto MDCK cell monolayers. Infected plates were incubated for 1 hr under cell culture conditions to allow for adherence and absorption. Plates were rocked every 15 min. Following incubation, plates were overlayed with 3 ml overlay medium (1 part liquid medium containing 1× MEM supplemented with l-glutamine (Gibco), HEPES solution (Gibco), 7.5% NaCHO3 solution (Gibco), penicillin-streptomycin-amphotericin B solution [Gibco), and 1 part 2.4% Avicel (FMC BioPolymer, Philadelphia, PA) in water with 1 μg/ml TPCK-trypsin). Plates were then incubated under cell culture conditions for 3 days. Following incubation, overlay was removed, and plates washed 2x with 1x PBS to remove residual overlay. Wells were fixed with 4% PFA for 15 min at room temperature. Following fixation, fixative was discarded, and wells were stained with crystal violet. Plaques were counted and viral titers calculated as described above.

### Statistics

All experiments were represented as a mean of independent replicates as indicated. Data were analyzed and plotted using Prism software (GraphPad, version 9.3.1). Statistical analyses were performed between groups using either unpaired t-test, ordinary one-way or two-way analysis of variance (ANOVA) as specified in individual figure captions with the indicated multiple comparisons tests.

## Supporting information

S1 Data All data used to generate figures. (XLSX)

S1 Fig Comparison of PB2_g1 potency against its reverse complementary version. A) Copy number of PB2 RNA in A549 cells infected with A/H1N1/California/04/09 MOI 0.01 and treated with Cas13 mRNA and the indicated guides using primer/probe Set 5. p = ** 0.01 and ****p< 0.0001 (Two-way ANOVA with Tukey’s multiple comparisons on log-transformed data). (B) Fold change of PB2 RNA levels normalized to NTCR condition for the data in part (A). p = *** 0.0002 and ****p< 0.0001 (Two-way ANOVA with Tukey’s multiple comparisons). In all parts, bars represent mean ± s.d. n = 6 per condition.
(TIF)

S2 Fig Absolute coverage of RNA-Seq reads for the all A/H1N1/California/04/09 segments. (A) PB2 segment (B) PB1 segment (C) HA segment (D) PA segment. (E) MP segment. (F) NP segment. (G)NA segment. (H) NS segment.
(TIF)

S3 Fig Normalized coverage of RNA-Seq reads for the all A/H1N1/California/04/09 segments. (A) PB1 segment (B) HA segment (C) PA segment (D) MP segment (E) NP segment. (F) NA segment. (G) NS segment.
(TIF)

S4 Fig Viral RNA counts for each A/H1N1/California/04/09 segment analyzed by RNA-Seq. Absolute viral RNA counts for each A/H1N1/California/04/09 segments in the indicated experimental conditions.
(TIF)

S5 Fig Screening of genome targeting guides across PB2. Raw fluorescence reads over time from the cell-free detection assay using the indicated crRNAs. Data are represented as mean of n = 3 per guide.
(TIF)

S6 Fig Delivery of Cas13 and guide PB2_g1 does not affect cell viability. (A) Percentage of live cells in the indicated experimental conditions measured upon treatment with Calcein AM and Ethidium Homodimer -1 at 494/517 nm. (B) Percentage of dead cells in the indicated experimental conditions measured upon treatment with Calcein AM and Ethidium Homodimer -1 at 528/617 nm.
(TIF)

S7 Fig Effect of Cas13’s enzymatic activity on viral KD. A) Copy number of PB2 RNA in A549 cells infected with A/H1N1/California/04/09 at the indicated MOIs and treated with Cas13 or GFP mRNA and guide PB2_g1 using primer/probe Set 5. *p* = * 0.0254 and ****p< 0.0001 (Two-way ANOVA with Tukey’s multiple comparisons on log-transformed data). (B) Direct comparisons of data in part (A) *p* = * 0.0053 (Two-way ANOVA on log-transformed data). (C) Fold change of PB2 RNA levels normalized to the NTCR condition for the data in part (A). ****p < 0.0001 (Two-way ANOVA with Tukey’s multiple comparisons). (D) Fold change of PB2 RNA levels normalized to the NTCR condition for the data in part (B). ****p < 0.0001 (Two-way ANOVA). In all parts bars represent mean ± s.d. n = 6 per condition.
(TIF)

S8 Fig Efficacy of guide PB2_g1 against Influenza A strains and across MOIs. (A) Copy number of PB2 RNA in A549 cells infected with A/Puerto Rico/8/1934 at the indicated MOIs and treated with Cas13 mRNA and guide PB2_g1 using a primer/probe set designed across the guide’s binding site (S1 Table). (B) Fold change of PB2 RNA levels normalized to the NTCR condition for the data in part (A). (C) Copy number of PB2 RNA in A549 cells infected with A/H1N1/California/07/09 at the indicated MOIs and treated with Cas13 mRNA and guide PB2_g1 using primer/probe Set 5. (D) Fold change of PB2 RNA levels normalized to the NTCR condition for the data in part (C). (E) Copy number of PB2 RNA in A549 cells infected with A/H3N2/Hong Kong/267/2019 at the indicated MOIs and treated with Cas13 mRNA and guide PB2_g1 using a primer/probe set designed across the guide’s binding site (S1 Table). (F) Fold change of PB2 RNA levels normalized to the NTCR condition for the data in part (E). (G) Copy number of PB2 RNA in A549 cells infected with A/WSN/33 at the indicated MOIs and treated with Cas13 mRNA and guide PB2_g1 a primer/probe set designed across the guide’s binding site (S1 Table). (H) Fold change of PB2 RNA levels normalized to the NTCR condition for the data in part (G). In all parts bars represent mean ± s.d. n = 6 per condition. **p 0.0069, *** *p*0.0009 and ****p < 0.0001 (Two-way ANOVA with Šídák’s multiple comparisons).
(TIF)

S9 Fig Efficacy of guide PB2_m4 against Influenza A strains and across MOIs. (A) Copy number of PB2 RNA in A549 cells infected with A/Puerto Rico/8/1934 at the indicated MOIs and treated with Cas13 mRNA and guide PB2_m4 using a primer/probe set designed across the guide’s binding site (S2 Table). (B) Fold change of PB2 RNA levels normalized to the NTCR condition for the data in part (A). (C) Copy number of PB2 RNA in A549 cells infected with A/WSN/33 at the indicated MOIs and treated with Cas13 mRNA and guide PB2_m4 using a primer/probe set designed across the guide’s binding site (S1 Table). (D) Fold change of PB2 RNA levels normalized to the NTCR condition for the data in part (C). In all parts bars represent mean ± s.d. n = 6 per condition. **p 0.0016, ***p 0.0003 and ****p < 0.0001 (Two-way ANOVA with Šídák’s multiple comparisons).
(TIF)

S10 Fig A/H1N1/California/04/09 infection characterization in hamster model and Cas13 mRNA + guide delivery evaluation. (A) Copy number of PB2 RNA in lungs of hamsters infected with A/H1N1/California/04/09 10^5^ PFU and isolated at the indicated days post infection (DPI). The bars represent mean ± s.d. n = 6 per condition. (B) Fold change of A/H1N1/California/04/09 PB2 levels in lungs of infected animals in part (A). The bars represent mean ± s.d. n = 6 per condition. (C) Lung viral titers for experiments in part (A). The bars represent mean ± s.d. n = 5 per condition. (D) Expression of a reporter Cas13a-NLuc mRNA formulated along with guide PB2_g1 with polymer P76 and delivered to the lungs by nebulization at the indicated doses. mRNA expression was measured as average radiance by IVIS imaging. n = 2 per condition. **p0.0017 (One-way ANOVA with Dunnett’s multiple comparisons on log-transformed data). (E) Fold change of TMEM41B mRNA in lungs of hamster treated with 300 μg of Cas13 mRNA + TMEM41B guide (S1 Table) formulated with polymer P76 and delivered by nebulization from control (untreated) animals. n = 6 per condition. ****p<0.0001 (One-way ANOVA with Dunnett’s multiple comparisons).
(TIF)

S11 Fig Nebulization of mRNA-expressed Cas13a and PB2_g1 as a treatment against Influenza H1N1 infection. (A) Fold change of PB2 RNA levels normalized to the virus only (Cal/04/09) condition for the data in Fig 6B. The bars represent mean ± s.d. n = 8 per condition. **p  0.0017 (Unpaired t test comparison on log transformed data). (B) Fold change of PB2 RNA levels normalized to the virus only (Cal/04/09) condition for the data in Fig 6E. The bars represent mean ± s.d. n = 8 per condition. *p  0.0186 (Unpaired t test comparison on log transformed data). (C) Fold change of PB2 RNA levels normalized to the virus only (Cal/04/09) condition for the data in Fig 6H. The bars represent mean ± s.d. n = 8 per condition. ****p < 0.0001 (Unpaired t test comparison on log transformed data).
(TIF)

S1 Table Sequences of unmodified or chemically modified crRNA guides, trRNA and reporter. (XLSX)

S2 Table Primer probe sets and standards for qRT-PCR. (XLSX)

S3 Table GAPDH CT fold change for the experiment described in Fig 1C. (XLSX)

S4 Table *p* values for the Tukey’s multiple comparisons test for the experiment described in Fig 1E. (XLSX)

S5 Table Raw data of absolute coverage of the whole PB2 gene segment in each treatment group normalized to the virus-only group. (XLSX)

S6 Table Raw data of absolute coverage of all IAV gene segments in each treatment group normalized to the virus-only group. (XLSX)

S7 Table *p* values for the Tukey’s multiple comparisons test for the experiment described in Fig 1H. (XLSX)

S8 Table *p* values for the Tukey’s multiple comparisons test for the experiment described in Fig 2D and 2E. (XLSX)

S9 Table ΔG values for the whole sequence and the target sequence of each genome targeting guide described in Fig 2A. (XLSX)

S10 Table *p* values for the Tukey’s multiple comparisons test for the experiment described in Fig 4E and 4F. (XLSX)

S11 Table Percentage of Identity and gaps between the sequence of guide PB2_g1 and available PB2 viral sequences using Blast (NCBI). (XLSX)

S12 Table *p* values for the Tukey’s multiple comparisons test for the experiment described in Fig 6B–6E. (XLSX)

S13 Table Changes in golden Syrian hamsters’ body weights for the experiment in S10 Fig. (XLSX)

S14 Table Changes in golden Syrian hamsters’ body weights for the experiment in Fig 7. (XLSX)

S15 Table 18S CT fold change for the experiments described in Fig 7. (XLSX)
