# Supplementary figures and images for: mRNA-encoded Cas13 treatment of Influenza via site-specific degradation of genomic RNA
Source: PLoS Pathog. 2024 Jul 5;20(7):e1012345. doi: 10.1371/journal.ppat.1012345 (PMC11253931; doi:10.1371/journal.ppat.1012345)

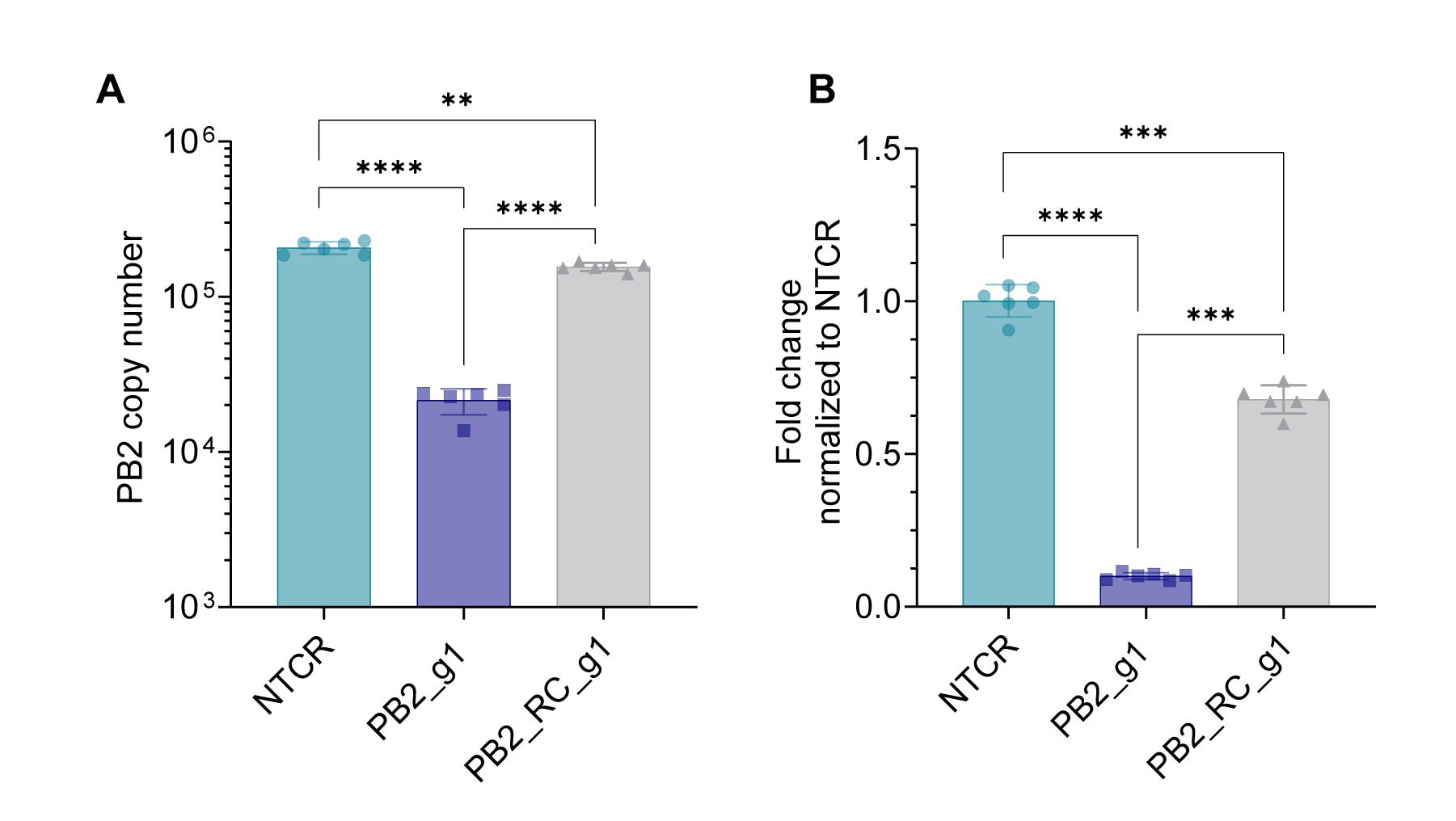

Supplement: S1 Fig — A) Copy number of PB2 RNA in A549 cells infected with A/H1N1/California/04/09 MOI 0.01 and treated with Cas13 mRNA and the indicated guides using primer/probe Set 5. p = ** 0.01 and ****p< 0.0001 (Two-way ANOVA with Tukey’s multiple comparisons on log-transformed data). (B) Fold change of PB2 RNA levels normalized to NTCR condition for the data in part (A). p = *** 0.0002 and ****p< 0.0001 (Two-way ANOVA with Tukey’s multiple comparisons). In all parts, bars represent mean ± s.d. n = 6 per condition. (TIF) [file ppat.1012345.s002.tif]

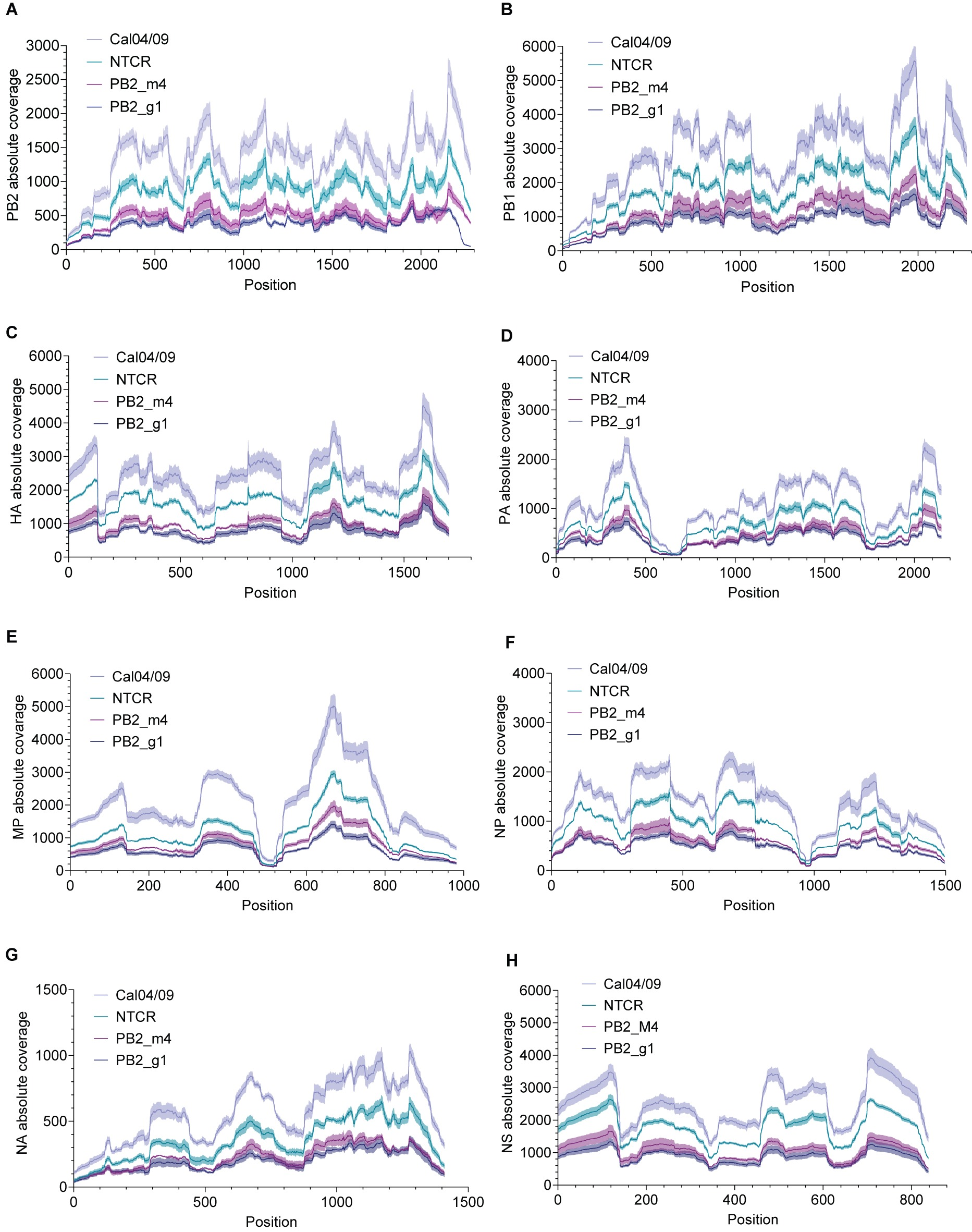

Supplement: S2 Fig — (A) PB2 segment (B) PB1 segment (C) HA segment (D) PA segment. (E) MP segment. (F) NP segment. (G)NA segment. (H) NS segment. (TIF) [file ppat.1012345.s003.tif]

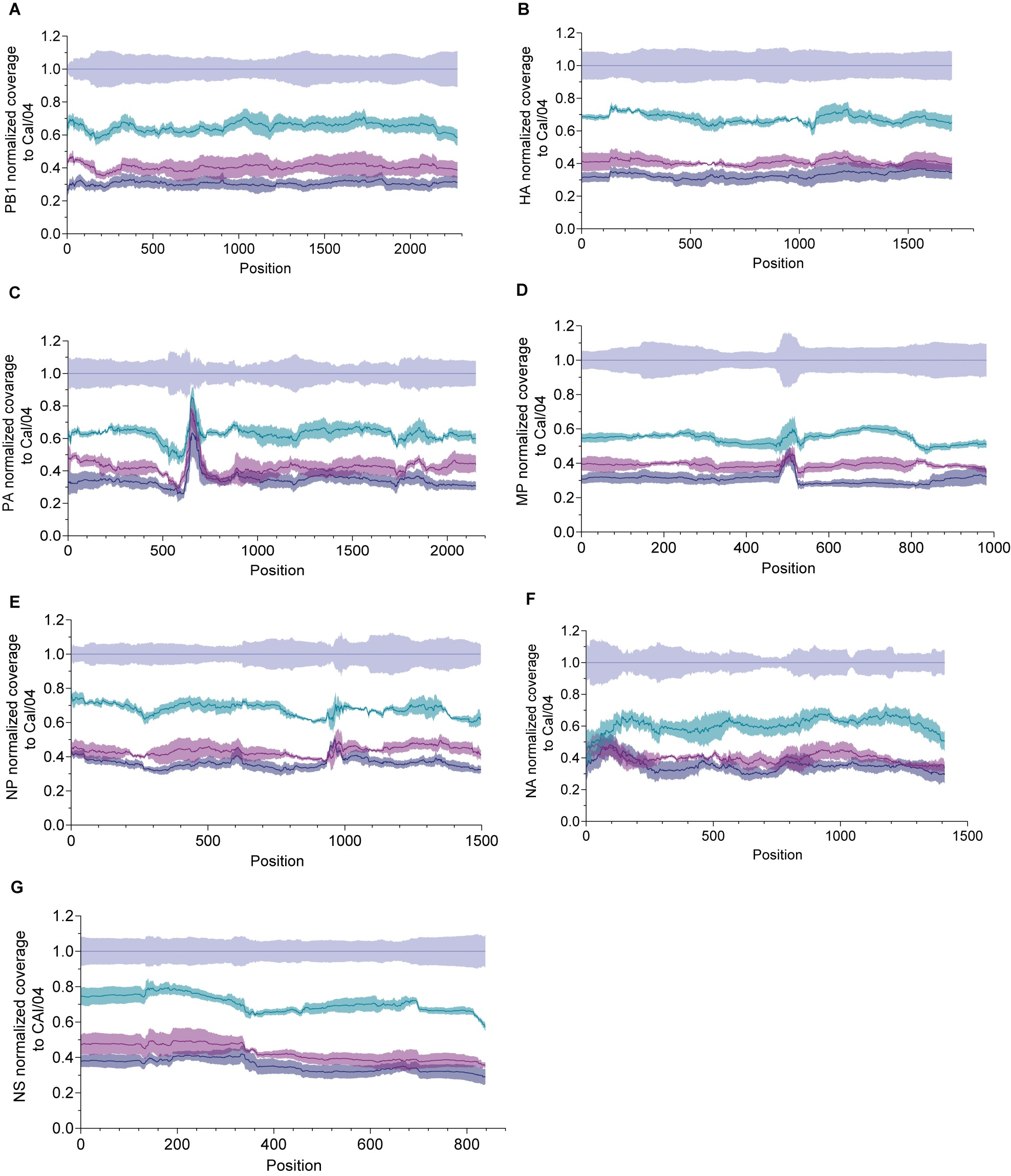

Supplement: S3 Fig — (A) PB1 segment (B) HA segment (C) PA segment (D) MP segment (E) NP segment. (F) NA segment. (G) NS segment. (TIF) [file ppat.1012345.s004.tif]

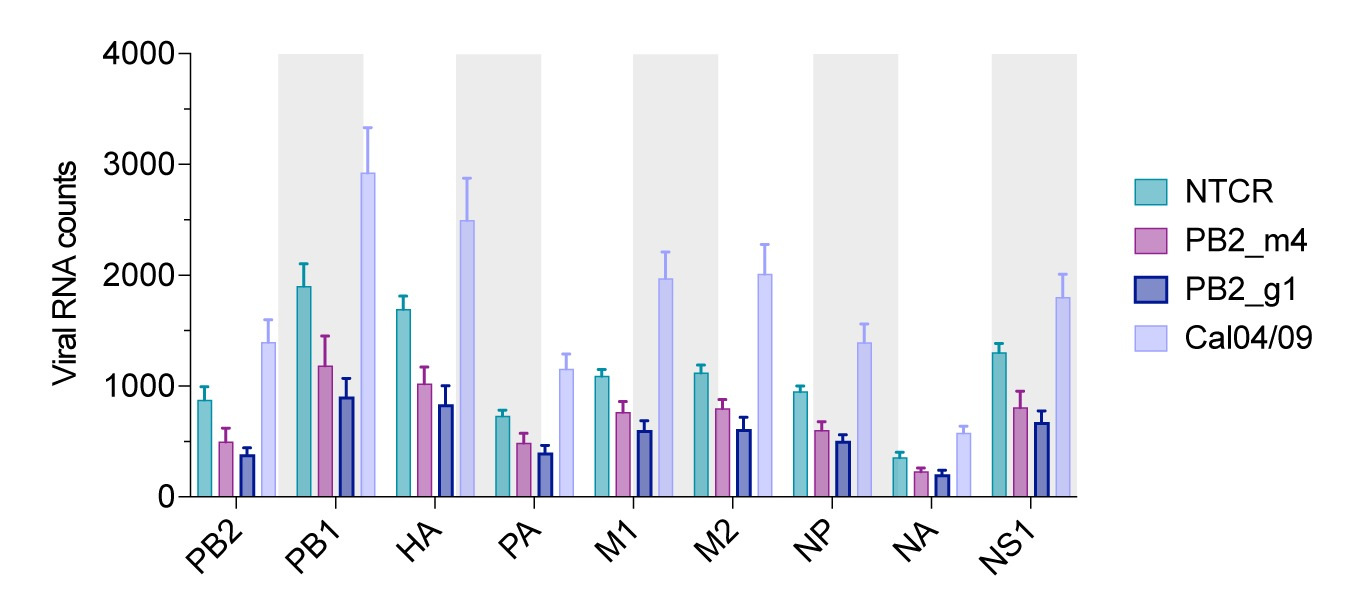

Supplement: S4 Fig — Absolute viral RNA counts for each A/H1N1/California/04/09 segments in the indicated experimental conditions. (TIF) [file ppat.1012345.s005.tif]

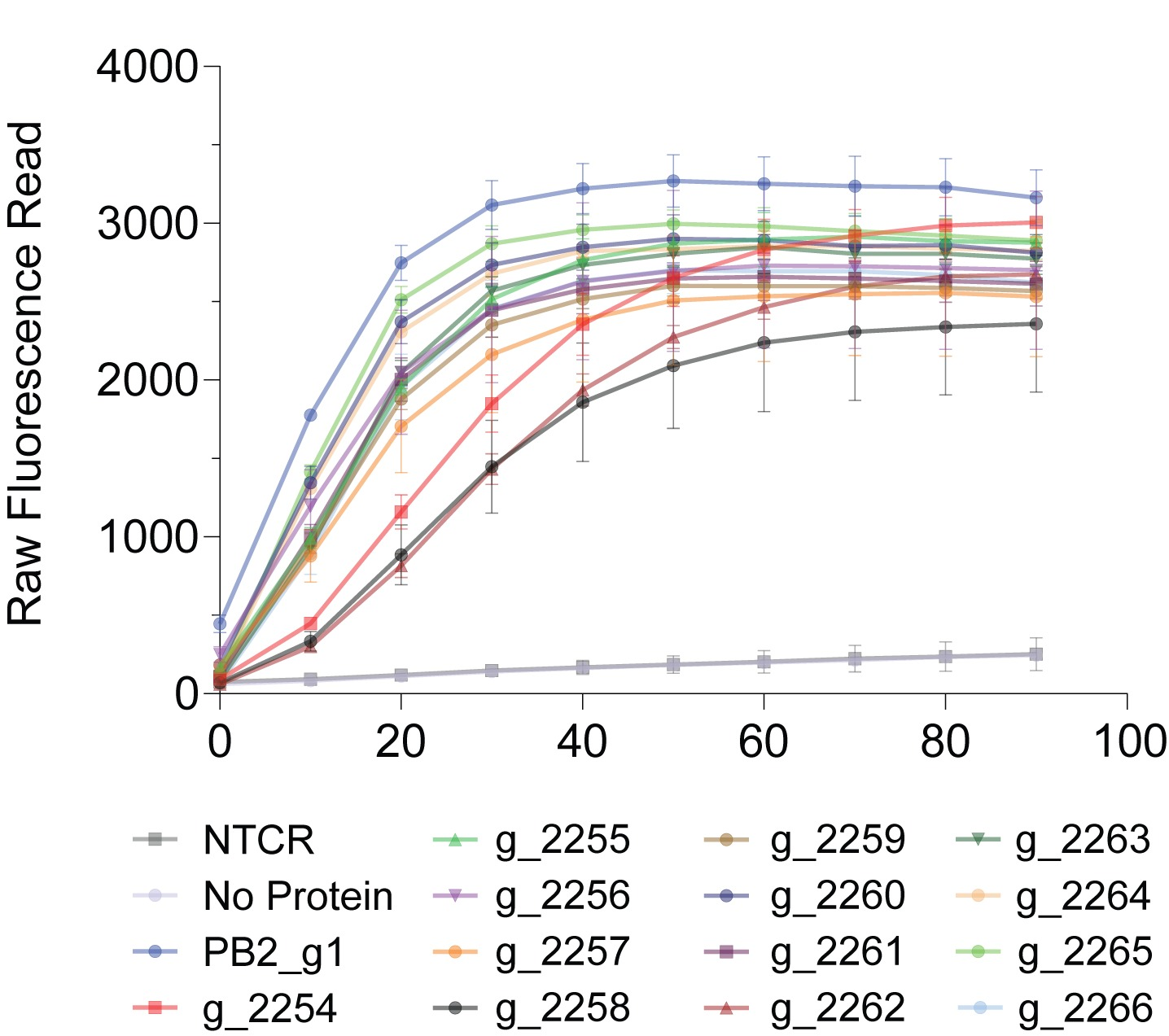

Supplement: S5 Fig — Raw fluorescence reads over time from the cell-free detection assay using the indicated crRNAs. Data are represented as mean of n = 3 per guide. (TIF) [file ppat.1012345.s006.tif]

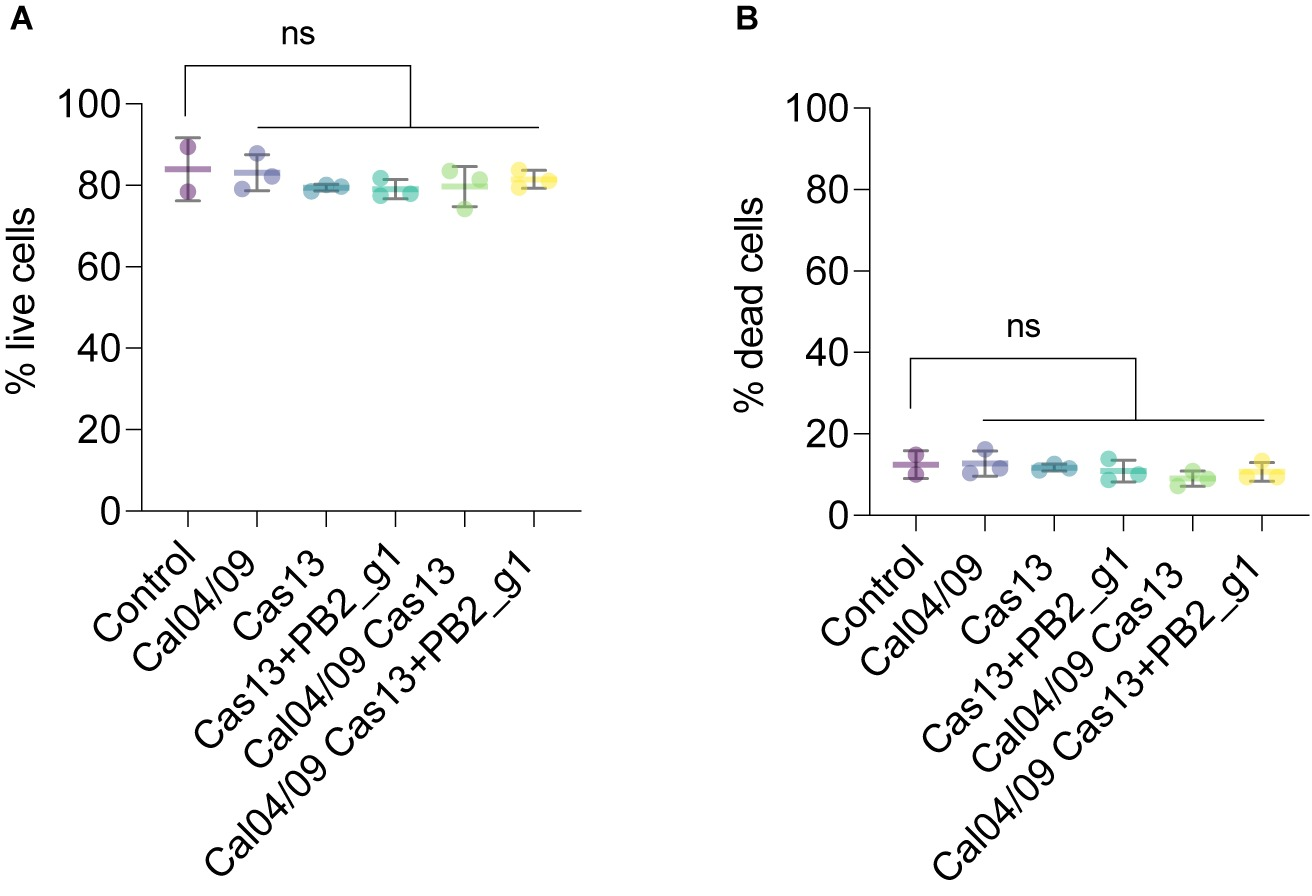

Supplement: S6 Fig — (A) Percentage of live cells in the indicated experimental conditions measured upon treatment with Calcein AM and Ethidium Homodimer -1 at 494/517 nm. (B) Percentage of dead cells in the indicated experimental conditions measured upon treatment with Calcein AM and Ethidium Homodimer -1 at 528/617 nm. (TIF) [file ppat.1012345.s007.tif]

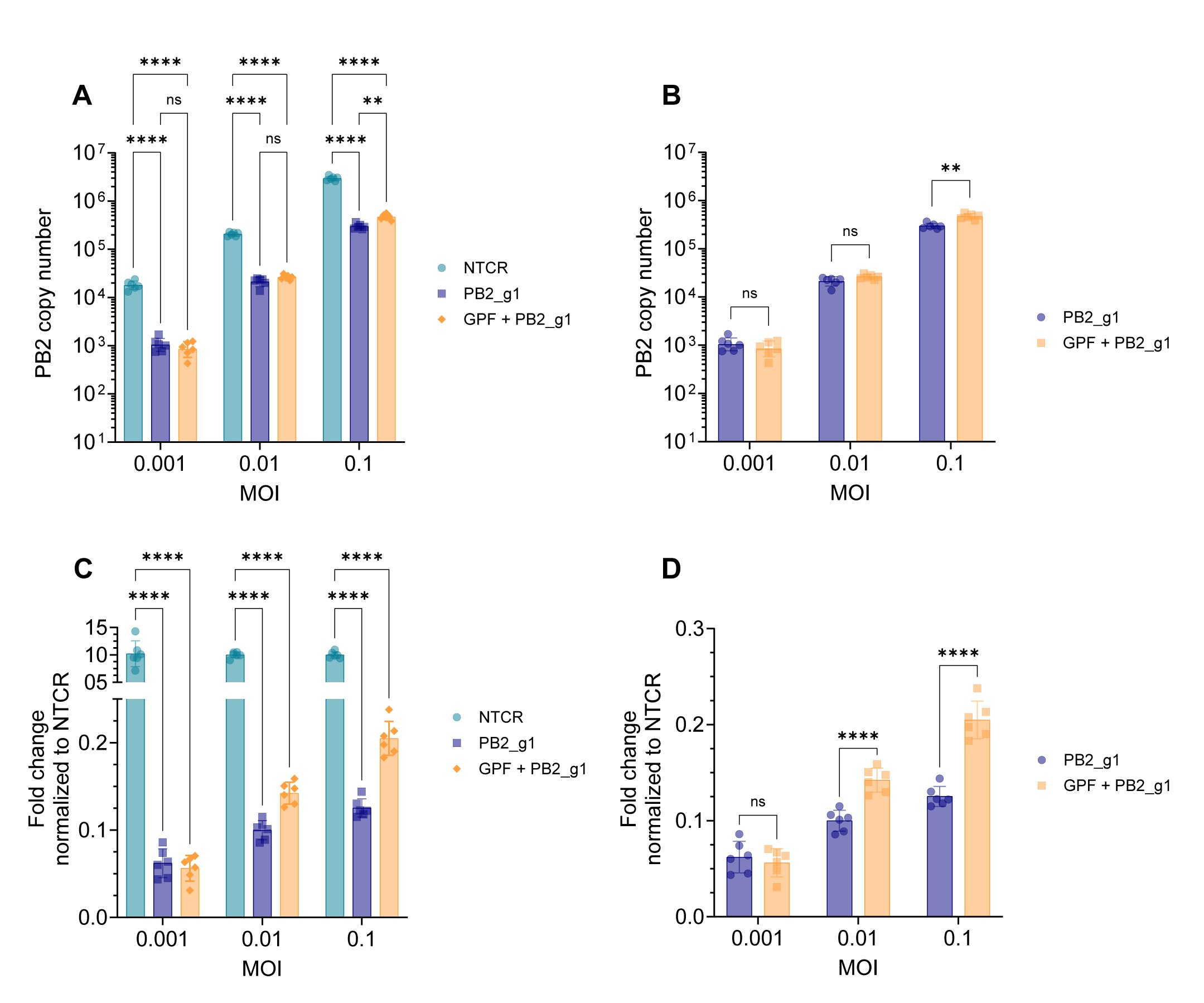

Supplement: S7 Fig — A) Copy number of PB2 RNA in A549 cells infected with A/H1N1/California/04/09 at the indicated MOIs and treated with Cas13 or GFP mRNA and guide PB2_g1 using primer/probe Set 5. p = * 0.0254 and ****p< 0.0001 (Two-way ANOVA with Tukey’s multiple comparisons on log-transformed data). (B) Direct comparisons of data in part (A) p = * 0.0053 (Two-way ANOVA on log-transformed data). (C) Fold change of PB2 RNA levels normalized to the NTCR condition for the data in part (A). ****p < 0.0001 (Two-way ANOVA with Tukey’s multiple comparisons). (D) Fold change of PB2 RNA levels normalized to the NTCR condition for the data in part (B). ****p < 0.0001 (Two-way ANOVA). In all parts bars represent mean ± s.d. n = 6 per condition. (TIF) [file ppat.1012345.s008.tif]

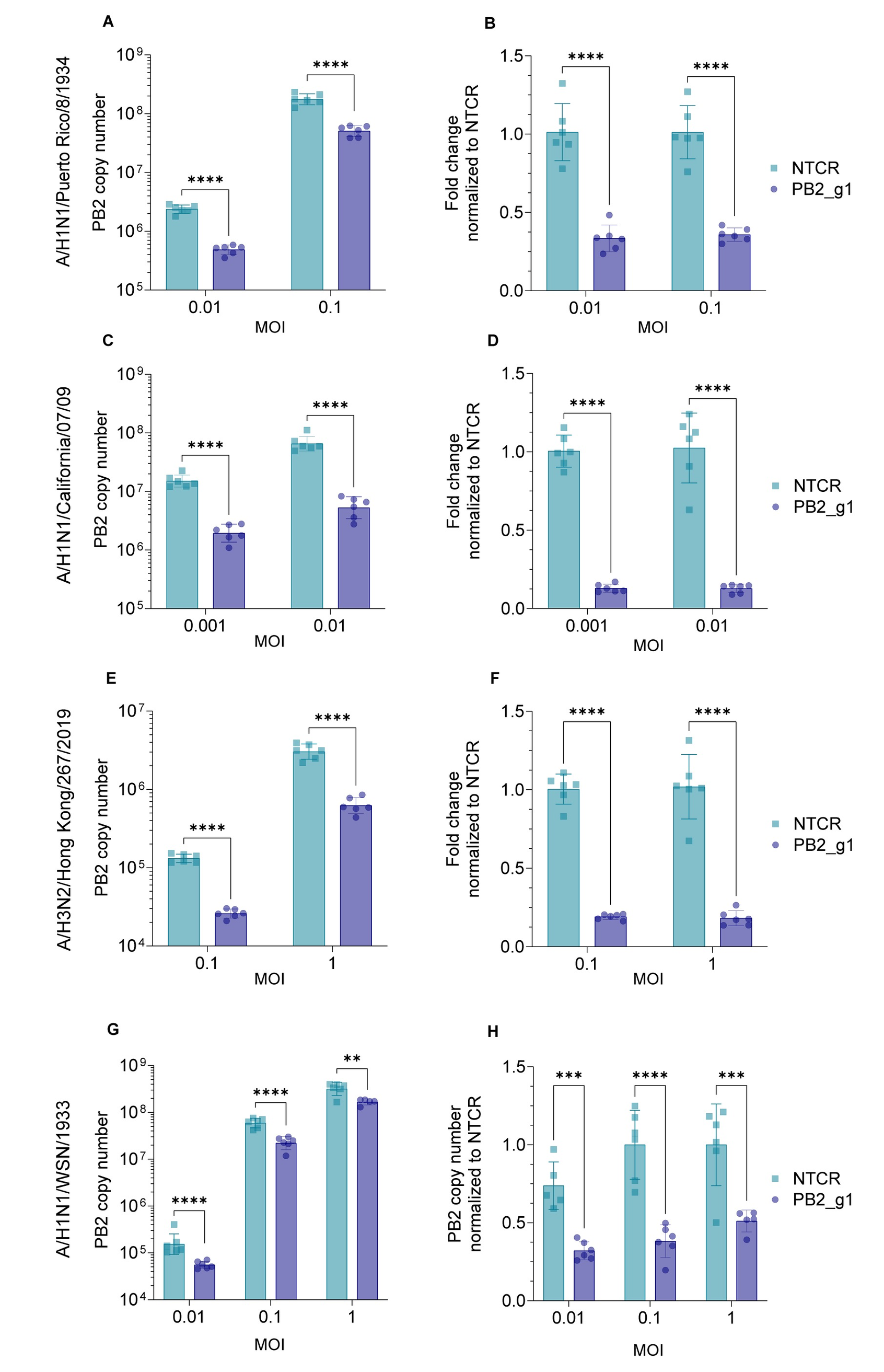

Supplement: S8 Fig — (A) Copy number of PB2 RNA in A549 cells infected with A/Puerto Rico/8/1934 at the indicated MOIs and treated with Cas13 mRNA and guide PB2_g1 using a primer/probe set designed across the guide’s binding site (S1 Table). (B) Fold change of PB2 RNA levels normalized to the NTCR condition for the data in part (A). (C) Copy number of PB2 RNA in A549 cells infected with A/H1N1/California/07/09 at the indicated MOIs and treated with Cas13 mRNA and guide PB2_g1 using primer/probe Set 5. (D) Fold change of PB2 RNA levels normalized to the NTCR condition for the data in part (C). (E) Copy number of PB2 RNA in A549 cells infected with A/H3N2/Hong Kong/267/2019 at the indicated MOIs and treated with Cas13 mRNA and guide PB2_g1 using a primer/probe set designed across the guide’s binding site (S1 Table). (F) Fold change of PB2 RNA levels normalized to the NTCR condition for the data in part (E). (G) Copy number of PB2 RNA in A549 cells infected with A/WSN/33 at the indicated MOIs and treated with Cas13 mRNA and guide PB2_g1 a primer/probe set designed across the guide’s binding site (S1 Table). (H) Fold change of PB2 RNA levels normalized to the NTCR condition for the data in part (G). In all parts bars represent mean ± s.d. n = 6 per condition. **p 0.0069, *** p0.0009 and ****p < 0.0001 (Two-way ANOVA with Šídák’s multiple comparisons). (TIF) [file ppat.1012345.s009.tif]

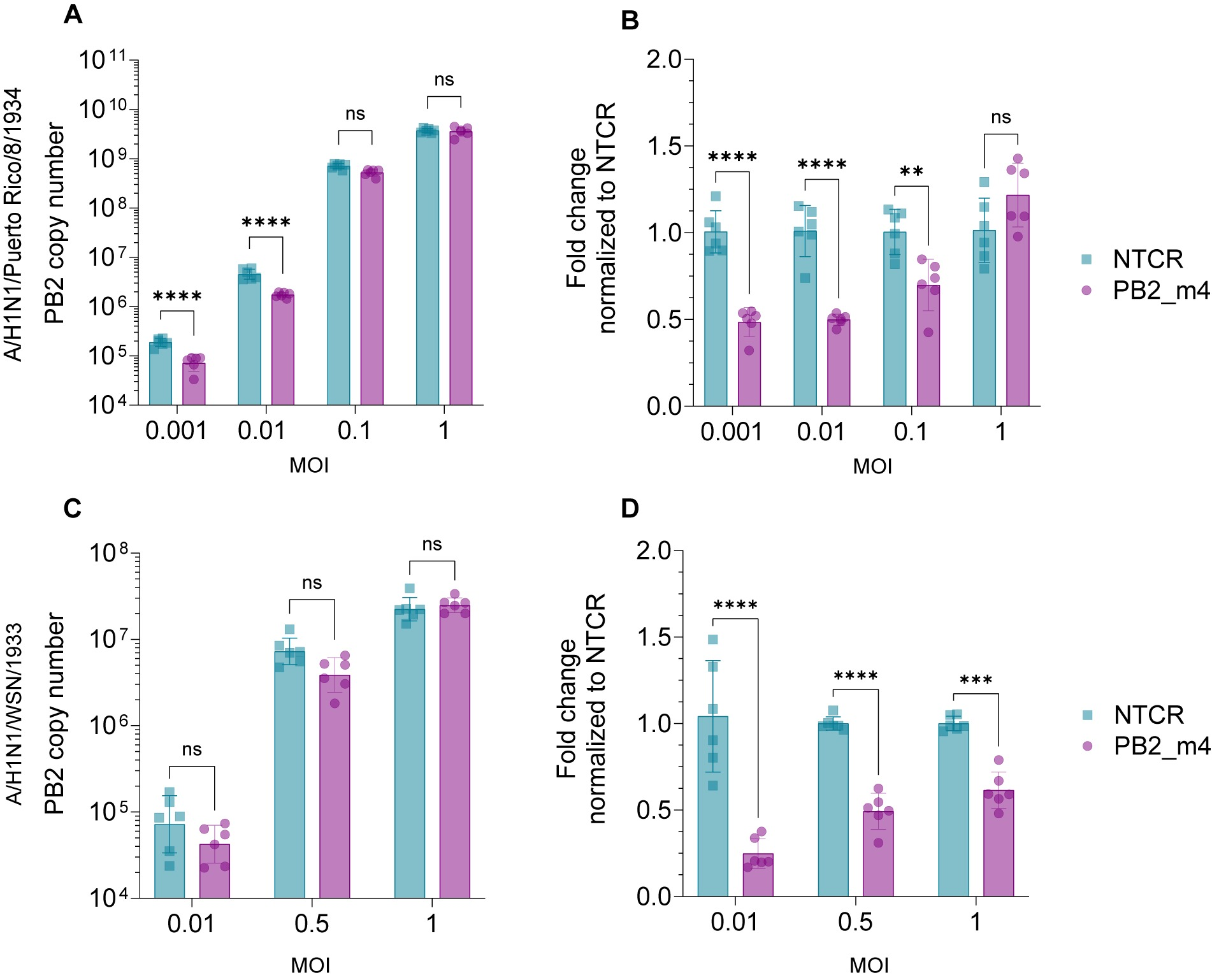

Supplement: S9 Fig — (A) Copy number of PB2 RNA in A549 cells infected with A/Puerto Rico/8/1934 at the indicated MOIs and treated with Cas13 mRNA and guide PB2_m4 using a primer/probe set designed across the guide’s binding site (S2 Table). (B) Fold change of PB2 RNA levels normalized to the NTCR condition for the data in part (A). (C) Copy number of PB2 RNA in A549 cells infected with A/WSN/33 at the indicated MOIs and treated with Cas13 mRNA and guide PB2_m4 using a primer/probe set designed across the guide’s binding site (S1 Table). (D) Fold change of PB2 RNA levels normalized to the NTCR condition for the data in part (C). In all parts bars represent mean ± s.d. n = 6 per condition. **p 0.0016, ***p 0.0003 and ****p < 0.0001 (Two-way ANOVA with Šídák’s multiple comparisons). (TIF) [file ppat.1012345.s010.tif]

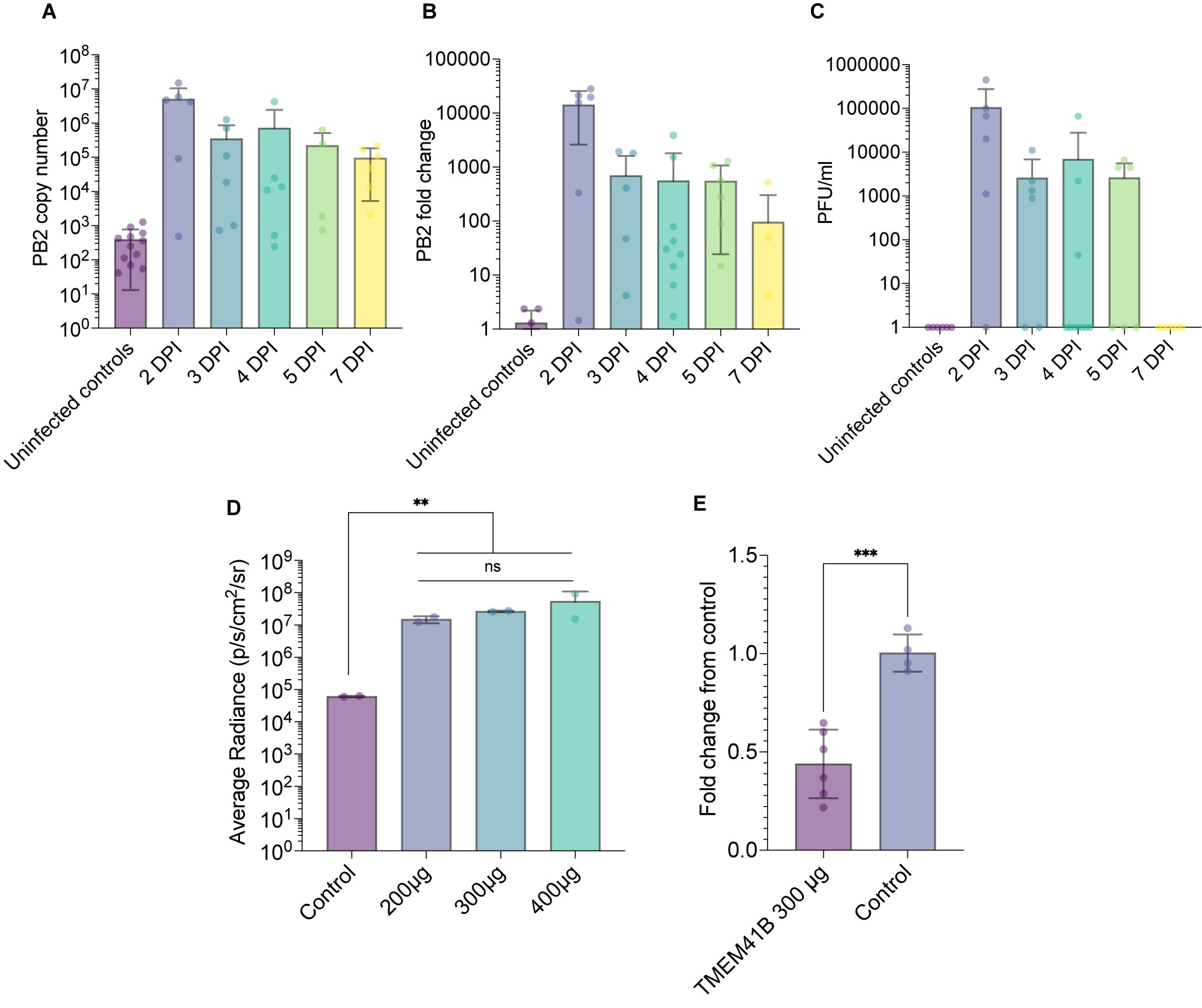

Supplement: S10 Fig — (A) Copy number of PB2 RNA in lungs of hamsters infected with A/H1N1/California/04/09 105 PFU and isolated at the indicated days post infection (DPI). The bars represent mean ± s.d. n = 6 per condition. (B) Fold change of A/H1N1/California/04/09 PB2 levels in lungs of infected animals in part (A). The bars represent mean ± s.d. n = 6 per condition. (C) Lung viral titers for experiments in part (A). The bars represent mean ± s.d. n = 5 per condition. (D) Expression of a reporter Cas13a-NLuc mRNA formulated along with guide PB2_g1 with polymer P76 and delivered to the lungs by nebulization at the indicated doses. mRNA expression was measured as average radiance by IVIS imaging. n = 2 per condition. **p0.0017 (One-way ANOVA with Dunnett’s multiple comparisons on log-transformed data). (E) Fold change of TMEM41B mRNA in lungs of hamster treated with 300 μg of Cas13 mRNA + TMEM41B guide (S1 Table) formulated with polymer P76 and delivered by nebulization from control (untreated) animals. n = 6 per condition. ****p<0.0001 (One-way ANOVA with Dunnett’s multiple comparisons). (TIF) [file ppat.1012345.s011.tif]

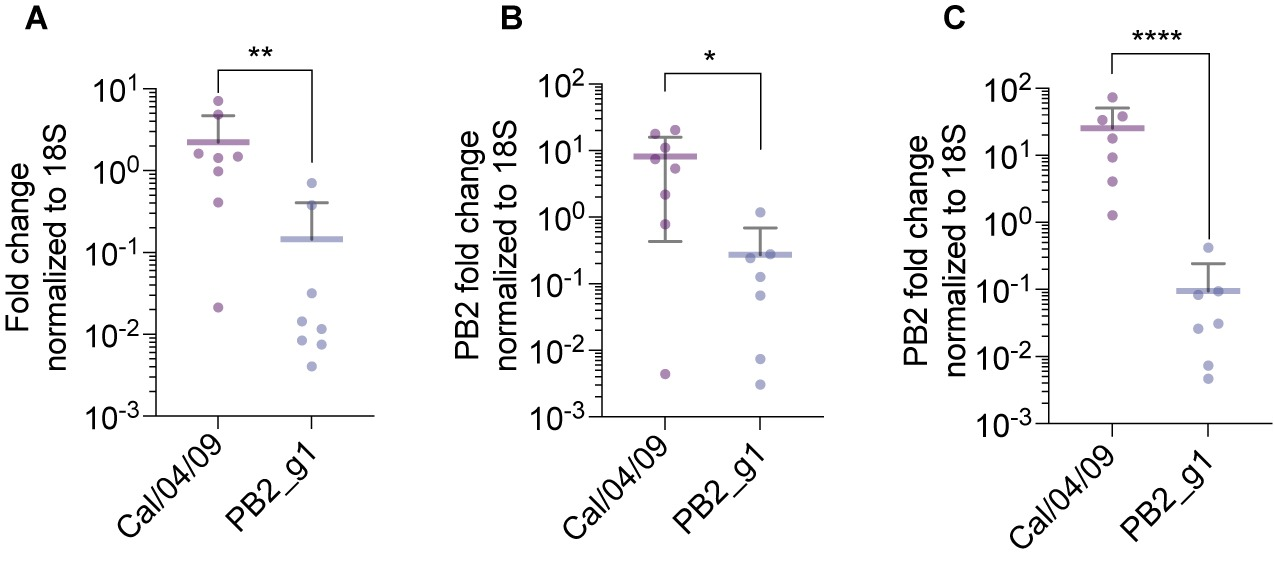

Supplement: S11 Fig — (A) Fold change of PB2 RNA levels normalized to the virus only (Cal/04/09) condition for the data in Fig 6B. The bars represent mean ± s.d. n = 8 per condition. **p 0.0017 (Unpaired t test comparison on log transformed data). (B) Fold change of PB2 RNA levels normalized to the virus only (Cal/04/09) condition for the data in Fig 6E. The bars represent mean ± s.d. n = 8 per condition. *p 0.0186 (Unpaired t test comparison on log transformed data). (C) Fold change of PB2 RNA levels normalized to the virus only (Cal/04/09) condition for the data in Fig 6H. The bars represent mean ± s.d. n = 8 per condition. ****p < 0.0001 (Unpaired t test comparison on log transformed data). (TIF) [file ppat.1012345.s012.tif]
